# Stream Macroinvertebrate Response Models for Bioassessment Metrics: Addressing the Issue of Spatial Scale

**DOI:** 10.1371/journal.pone.0090944

**Published:** 2014-03-27

**Authors:** Ian R. Waite, Jonathan G. Kennen, Jason T. May, Larry R. Brown, Thomas F. Cuffney, Kimberly A. Jones, James L. Orlando

**Affiliations:** 1 U.S. Geological Survey, Portland, Oregon, United States of America; 2 U.S. Geological Survey, West Trenton, New Jersey, United States of America; 3 U.S. Geological Survey, Sacramento, California, United States of America; 4 U.S. Geological Survey, Raleigh, North Carolina, United States of America; 5 U.S. Geological Survey, Salt Lake City, Utah, United States of America; Northwest Fisheries Science Center, NOAA Fisheries, United States of America

## Abstract

We developed independent predictive disturbance models for a full regional data set and four individual ecoregions (Full Region vs. Individual Ecoregion models) to evaluate effects of spatial scale on the assessment of human landscape modification, on predicted response of stream biota, and the effect of other possible confounding factors, such as watershed size and elevation, on model performance. We selected macroinvertebrate sampling sites for model development (n = 591) and validation (n = 467) that met strict screening criteria from four proximal ecoregions in the northeastern U.S.: North Central Appalachians, Ridge and Valley, Northeastern Highlands, and Northern Piedmont. Models were developed using boosted regression tree (BRT) techniques for four macroinvertebrate metrics; results were compared among ecoregions and metrics. Comparing within a region but across the four macroinvertebrate metrics, the average richness of tolerant taxa (RichTOL) had the highest R^2^ for BRT models. Across the four metrics, final BRT models had between four and seven explanatory variables and always included a variable related to urbanization (e.g., population density, percent urban, or percent manmade channels), and either a measure of hydrologic runoff (e.g., minimum April, average December, or maximum monthly runoff) and(or) a natural landscape factor (e.g., riparian slope, precipitation, and elevation), or a measure of riparian disturbance. Contrary to our expectations, Full Region models explained nearly as much variance in the macroinvertebrate data as Individual Ecoregion models, and taking into account watershed size or elevation did not appear to improve model performance. As a result, it may be advantageous for bioassessment programs to develop large regional models as a preliminary assessment of overall disturbance conditions as long as the range in natural landscape variability is not excessive.

## Introduction

Understanding the effects of human land use modification on stream biota, the processes that cause these effects, and the various spatial and temporal scales at which these effects and processes operate are fundamental goals of bioassessment in stream ecology. The use of models has increased markedly in the past decade in all areas of ecology partly to address the issues stated above. In addition, major advances have been made in conceptual models and statistical techniques [1]–[7], which, in turn, help practitioners derive response models that better support the needs of bioassessment programs. Models provide a useful framework for testing hypotheses, determining potential direct and indirect linkages, and directing where further research is needed. The expansion and application of multivariate models in stream ecology are helping to address these issues and hopefully will lead to a broader understanding of ecological and anthropogenic pathways and responses [3],[4],[8].

Spatial scale is important in understanding ecosystem processes and species distributions; as such, scale is an important consideration in ecological research [9]–[11]. At the same time, managers often need decision-making tools that can be applied to as large a region as possible. Cuffney et al. [6] showed that responses of algal and macroinvertebrate metrics to urbanization varied by taxon, geographic region, and antecedent land use. Stevenson et al. [12] modeled algal biomass and found a positive correlation with nutrients in two distinct regions, yet the amount of variation explained differed by nutrient concentration and region. Potapova and Charles [13] suggested that algal indicators and optima were improved if developed regionally rather than nationally. Ode et al. [14] found that macroinvertebrate indices developed at large regional scales, such as the western U.S., had lower precision in California than California-based indices. They found that the larger scale indices were influenced by two natural gradients that did not affect the statewide indices. Seelbach et al. [15], on the other hand, found that regions too large can sometimes give misleading results when strong natural gradients at larger scales are mismatched with ecological-scale responses. For example, their streamflow models across three states in the midwestern U.S. showed that more rainfall in the southern portion of their region created lower stream baseflows (a nonsensical relationship) and that higher northern baseflows were the result of very permeable glacial deposits that are variable across smaller scales that were not accounted for by the large scale models [15]. Overall, national or regional scale models are likely to focus on large scale natural landscape effects such as climate, typography, elevation, and geology as the primary discriminating variables, with disturbance variables such as land use, nutrients, sediments, and contaminants as secondary variables. However, current ecosystem theory indicates that models at smaller scales should allow for more insight and interpretation of disturbance related processes or mechanisms that are likely to operate at smaller watershed and site specific scales [5],[6],[16],[17].

Research documenting the effects of land-use change on stream biota indicates that as the amount of urban and(or) agricultural land use in the watershed increases, individual biological metrics and multimetric indices (e.g., Index of Biotic Integrity – IBI) that reflect compositional changes in sensitive species generally decrease [10],[11],[18],[19],[20]. However, a better understanding of the effect of spatial scale on disturbance processes and the subsequent effect on modeling performance is paramount for developing models that better support bioassessment efforts and regulatory application [21]. Waite et al. [11] developed macroinvertebrate response models for three regions in the western U.S. The best multiple linear regression (MLR) models from each individual region required only two or three explanatory variables to model macroinvertebrate metrics. In each region, their best model contained some measure of urban and(or) agricultural land use, yet often the model was improved by including a natural landscape factor such as mean annual precipitation or mean watershed slope. Brown et al. [22] were also able to develop strong models using modeling techniques such as MLR and boosted regression trees (BRT). They modeled a macroinvertebrate index of biotic integrity (BIBI) across a gradient of urbanized streams in southern California using MLR models; however, overall model prediction and performance was generally improved by using BRT.

Regression trees are one type of modeling technique within the commonly used classification and regression tree (CART) or decision tree family (e.g., [23]–[25]). These modeling techniques have some highly desirable properties, including the ability to handle categorical and censored data and non-normal distributions, and they can model complex interactions simply [26]. De'ath [26] and Elith et al. [27] showed that the more flexible BRT models outperform general linear models (GLM) and general additive models (GAM) in variable selection and predictive ability (higher R^2^ and lower error) and can handle sharp discontinuities in data that are difficult for the other methods. Aertsen et al. [28] also showed that BRT outperformed most modeling techniques (MLR, GLM, GAM, and CART), with the exception of artificial neural networks, which the authors penalized for being complex and non-transparent.

The primary goal of this paper is to evaluate the influence of scale on predictive macroinvertebrate response models developed across four proximal ecoregions. We compare the overall performance (i.e., R^2^ and cross-validation R^2^) of models for predicting the responses of common macroinvertebrate metrics (e.g., EPT Richness, Tolerance, and Non-Insects) developed using BRT techniques. Potential explanatory variables in the models include common landscape-based disturbance variables such as land use, road and population density, riparian canopy, infrastructure, and hydrologic runoff as well as natural landscape factors such as climate, slope, elevation, and soil infiltration. Our objectives are to test whether (1) the small more region specific scales (individual ecoregions) will allow for better performing models compared to the larger full regional scale models and (2) the ecoregion scaled models will highlight more region-specific disturbance-related explanatory variables (e.g., hydrologic runoff and region specific landscape disturbance variables) compared to higher inclusion of more large scale natural setting variables in the full region models.

In addition, we evaluate whether elevation and watershed size classes are better predictors of common macroinvertebrate metrics than the Full Region model and hypothesize that BRT models developed for each of three a-priori watershed size classes will produce stronger predictive models than that for the full region because stream size and location along the gradient from headwaters to the mouth influences the type and distribution of aquatic fauna [29], a similar logic for the elevation classes. In this context, hydrologic variability and, subsequently, stream biota are highly dependent on their longitudinal location along the continuum; therefore, we would expect that models with greater predictive power would be developed, for example, in small low order streams (streams <27 km^2^) compared to models of large watershed streams or models developed for the Full Region that blends all stream sizes.

## Methods

### Description of modeling regions

Macroinvertebrate data aggregated across four proximal level III ecoregions—the North Central Appalachians, Ridge and Valley, Northeastern Highlands, and Northern Piedmont, located in the northeastern U.S.—were used in our analysis. These four ecoregions have broad similarities in some features such as climate and forest land cover, yet there are stark differences among some of these regions in basic physiography, climate, and agriculture and urban land use. Mean and range of select environmental variables for each region are provided (variable list and definitions: Table 1, mean and ranges: Table 2).

**Table 1:** Description, variable code and definition of explanatory environmental (landscape, riparian and habitat) and response (invertebrate metrics) variables used for model development.

Description	Variable Code	Definition
Explanatory Variables		
Watershed Variables		
Percent Agricultural Landuse	Ag	Percent watershed area in agricultural landuse (NLCD 2001 category 81, 82)
Percent Urban Landuse	Urban	Percent watershed area in urban landuse (NLCD 2001 categories 21, 22, 23, and 24)
Sum of Percent Ag + Urban	Ag+Urb	Sum of percent watershed area in urban (NLCD 2001 categories 21, 22, 23, and 24) and agricultural (NLCD 81, 82) landuse
Percent Forest	Forest	Percent watershed area in forest landuse (NLCD 2001 categories 41, 42, 43)
Percent Wetland	Wetland	Percent watershed area in wetland land cover (NLCD 2001 category 90 and 95)
Road Density	Rd.Density	Road density in watershed = Road length (km)/watershed area (km^2^)
Mean Population Density	Pop.Density	Watershed mean population density based on 2000 census (#/km^2^)
Dam Density	Dam Density	Density of dams in watershed = Number of dams/watershed area (km^2^)
Percent Manmade Channel	ManMadeChan	Percentage of linear water features in stream buffer which are manmade
Riparian Variables		
Percent Agricultural Landuse	Rip.AG	Percent buffer area in agricultural landuse (NLCD 2001 category 81, 82) in riparian buffer
Percent Urban Landuse	Rip.Urban	Percent buffer area in urban landuse (NLCD 2001 categories 21, 22, 23, and 24) in riparian buffer
Sum of Percent Ag + Urban	Rip.Ag+Urb	Sum of percent buffer area in urban (NLCD 2001 categories 21, 22, 23, and 24) and agricultural (NLCD 81, 82) landuse in riparian buffer
Percent Forest	Rip.Forest	Percent buffer area in forest landuse (NLCD 2001 categories 41, 42, 43) in riparian buffer
Mean Tree Canopy Cover	Rip.Canopy	Percent canopy cover (NLCD 2001 Percent Tree Canopy dataset; 30 m pixel) in riparian buffer
Road Density	Rip.Rd.Dens	Road density in buffer = Road length (km)/riparian buffer area (km^2^) in
Mean Population Density	Rip.Pop.Dens	Buffer area mean population density based on 2000 census (#/km^2^)
Natural Landscape Variables		
Watershed Mean Elevation	Mn.Elev	Mean watershed elevation (m)
Watershed Mean Slope Percent	Slope	Mean percent watershed slope (%)
Mean Annual Precipitation	Mn.Ann.Precip	Mean annual precipitation (mm)
Riparian Mean Slope Percent	Rip.Slope	Mean percent riparian buffer slope (%)
Riparian Maximum Elevation	Rip.Max.Elev	Maximum riparian buffer elevation (m)
Soil Infiltration Rate B	Soil Infiltration B	Area of stream buffer having soils with moderate infiltration rates (From NRCS, STATSGO database) (km^2^)
Soil Infiltration Rate C	Soil Infiltration C	Area of stream buffer having soils with slow infiltration rates (From NRCS, STATSGO database) (km^2^)
Soil Infiltration Rate D	Soil Infiltration D	Area of stream buffer having soils with very slow infiltration rates (From NRCS, STATSGO database) (km^2^)
Hydrologic Runoff Variables		
Average Monthly Coefficient of Variation	Ave_MonthCV	Coefficient of variation of average monthly runoff values for 2001
Maximum Monthly Runoff	Max_Monthly	Maximum monthly runoff for 2001 (mm)
Maximum Monthly Coefficient of Variation	Max_MonthCV	Coefficient of variation of maximum monthly runoff values for 2001
Maximum Runoff for January Months	Max_January	Maximum runoff for January 2001 (mm)
Average March Runoff	Ave_March	Average runoff for March 2001 (mm)
Maximum March Runoff	Max_March	Maximum runoff for March 2001 (mm)
Minimum Runoff for April	Min_April	Minimum runoff for April 2001 (mm)
Maximum Runoff for April	Max_April	Maxmum runoff for April 2001 (mm)
Average Spring Runoff	Ave_Spring	Average runoff for April and May 2001 (mm)
Maximum Runoff for May	Max_May	Maximum runoff for May2001 (mm)
Maximum Runoff for July	Ave_July	Maximum runoff for July 2001 (mm)
Maximum Runoff for November	Max_Nov	Maximum runoff for November 2001 (mm)
Average December Runoff	Ave_Dec	Average runoff for December 2001 (mm)
Response Variables: Invertebrate Metrics		
Ephemeroptera, Plecoptera, and Trichoptera Richness	EPTR	Richness composed of Mayflies, Stoneflies and Caddisflies for a sample
Average Tolerance of Taxa in a Sample	RichTOL	Average USEPA tolerance values for sample based on richness
Intolerant Richness	INTOL_RICH	Number of USEPA intolerance (0 – 4 values) taxa
Noninsect Richness	NonInsectR	Total richness composed on noninsects

All variables listed were initially considered for inclusion in boosted regression tree (BRT) models. NLCD–National Land Cover Dataset, NRCS–National Resource Conservation Service, STATSGO–State Soil Geographic data base.

**Table 2:** Summary of select environmental variables by region, numeric variables presented as average values, range in parentheses.*

Region	Urban (%)	Agriculture (%)	Population Density (#/km^2^)	Manmade Channels (%)	Ave_Spring Runoff (mm)	Mean Annual Precipitation (mm)	Mean Slope (%)
**Full Region**	**13**	**20**	**421**	**2.1**	**60**	**1190**	**7**
n = 591	(0 – 94)	(0 – 97)	(0 – 13300)	(0 – 40)	(31 – 116)	(900 – 1610)	(0.4 – 18.8)
**North Central Appalachians**	**4**	**7**	**52**	**0.7**	**63**	**1140**	**8.1**
n = 167	(0 – 37)	(0 – 50)	(0 – 390)	(0 – 17)	(52 – 87)	(900 – 1450)	(1.7 – 18.8)
**Ridge and Valley**	**11**	**29**	**215**	**2.0**	**55**	**1170**	**7.3**
n = 152	(0 – 88)	(0 – 75)	(0 – 2100)	(0 – 40)	(34 – 87)	(1010 – 1330)	(1.6 – 13.0)
**Northeastern Highlands**	**10**	**12**	**257**	**2.5**	**74**	**1240**	**8.0**
n = 139	(0 – 56)	(0 – 57)	(0 – 1800)	(0 – 34)	(44 – 116)	(990 – 1610)	(3.3 – 17.0)
**Northern Piedmont**	**29**	**34**	**1290**	**3.4**	**45**	**1230**	**3.5**
n = 133	(1 – 94)	(0 – 97)	(70 – 13300)	(0 – 32)	(31 – 58)	(1060 – 1350)	(0.4 – 8.3)

*Ave_Spring equal to the average runoff for the spring months (April-May).

The North Central Appalachians ecoregion is a partially glaciated and forested plateau of horizontally bedded shale, siltstone, sandstone, coal, and conglomerate that is punctuated by high hills and low mountains [30]. The climate can be characterized as continental, with cool summers and cold winters. The average annual precipitation ranges from 900 to 1,450 mm per year. The Ridge and Valley ecoregion is a series of alternating elongated forested ridges and narrow agricultural valleys created by tightly folded and intensely faulted bedrock composed chiefly of shale, limestone, sandstone, and dolomite under a veneer of unconsolidated materials [31], [32]. Elevations typically range from 91 to 1,220 m and precipitation ranges from 1,010 to 1,330 mm, with 20 to 30 percent of the annual precipitation falling as snow. Stream patterns are generally trellis-shaped and reflect the regular folding of the geomorphology with high-gradient streams along ridge slopes, and gentle gradient, meandering streams in the valleys. Land cover is predominantly forests (about 56 percent) with a mixture of agricultural, developed, and managed lands (e.g., state parks and wild and scenic rivers).

The Northeastern Highlands is a rugged, high elevation forested region characterized by a series of northeast-trending ridges and valleys with elevations ranging from about 43 m to over 425 m on the highest ridges. The entire physiographic area is noteworthy as a sparsely populated corridor of forests, wetlands, and grasslands of regional importance to migratory birds and many other plant and animal communities all within close proximity of the greater New York City metropolitan area of over 20 million people [33]. The forest is dominated by upland hardwood forest types on the ridges and valley slopes, and forested wetlands in the valleys. Annual average rainfall varies from 990 to 1,610 mm, with significantly higher amounts in mountainous areas. The Northern Piedmont ecoregion trends northeast to southwest covering parts of northeastern New Jersey and southeastern Pennsylvania and consists of northwestward-dipping sedimentary rocks that form rounded hills, irregular plains, and open valleys [3]. Elevations range from about 99 m on limestone to 396 m on more resistant metamorphic rock, with some isolated, rocky and higher hills or ridges. The climate includes moderate winters and warm, humid summers (i.e., humid continental) with more than 1,000 mm of precipitation falling in an average year. Land use varies, ranging from busy urban and suburban areas, to intensely farmed and densely settled locales, to relatively quiet pastoral places, which forms a mosaic of agricultural, forest, and developed lands, but the mixture varies, depending on local conditions.

The Northern Piedmont (N_Pied) has the highest mean percent urban and agriculture (29 and 34%, respectively) among the four ecoregions, the Ridge and Valley (R&V) has the second highest (11 and 29%, respectively) (Table 2). The North Central Appalachians (NC_App) has the lowest percentage of urban and agriculture (4 and 7%, respectively), however, the proportion of urban and agriculture in the Northeastern Highlands (NE_High) is only slightly higher (10 and 12%, respectively). In general, these two ecoregions tend to be slightly less developed than the N_Pied and R&V. The N_Pied also has slightly more than five times the average population density than the next highest ecoregion (i.e., NE_High) and has the highest percent manmade channels, yet the lowest average spring runoff and mean watershed slope (Table 2). Mean annual precipitation across all ecoregions is generally similar and ranges from 1140 to 1240 mm (Table 2). Percent slope is highest for NC_App and lowest for N_Pied.

### Data Aggregation and Landscape Analysis

**Figure 1:**
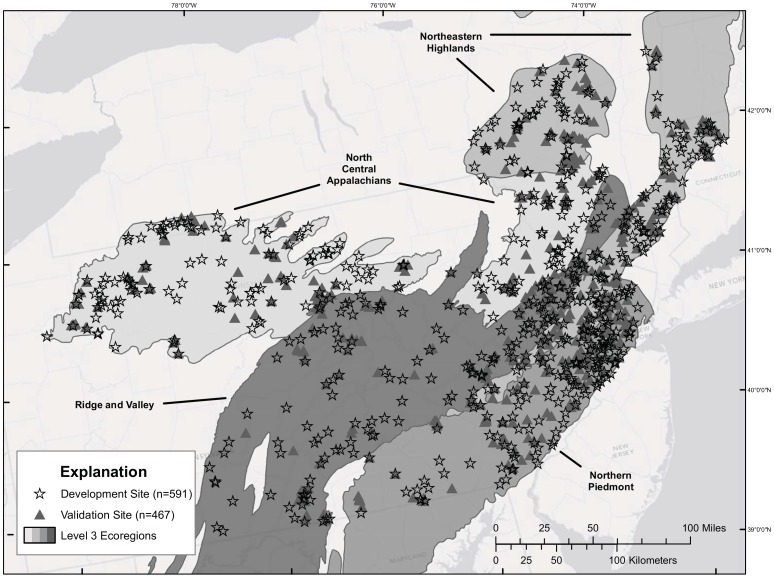
Map showing the stream sites used for model development (stars) and model validation (triangles). Sites used for model development (stars) (n = 591) and model validation (triangles) (n = 467) are evenly spread across the four ecoregions used in this study (N.C. Appalachian, Ridge and Valley, N.E. Highlands and N. Piedmont).

ArcGIS, ArcMap 9.2 (Environmental Systems Research Institute, Redlands, CA), was used to create, interpret, and analyze spatial data sets representing sampling site locations, watershed area and size class, riparian zones, and potential sources of anthropogenic disturbance for all watersheds located throughout the study area (Supplemental File: Appendix S1). Site location data sets were obtained from federal and state agencies and partners, including the U.S. Geological Survey, Connecticut Department of Environmental Protection (CTDEP), New Jersey Department of Environmental Protection (NJDEP), New York Department of Environmental Conservation (NYDEC), and Pennsylvania Department of Environment Protection (PADEP). These data sets were combined and standardized to create one seamless data set with over 10,000 sample sites. The data were then screened and censored according to the following criteria: (1) sampling methodology, (2) sampling season and year (we retained only samples taken between May and October from 1996 to 2009 that best coincide with the 2001 land use data), (3) upstream watershed area of > 8.0 and <777.0 km^2^, and (4) watersheds not nested (i.e., not linked by downstream flow). In addition, sites that represented multiple sampling events in the same location were removed. In total, 1058 sites met the criteria and were used either as model development (n = 591) or validation (n = 467) sites (Figure 1). The development data set included sites within the four contiguous ecoregions: North Central Appalachians (n = 167), Ridge and Valley (n = 152), Northeastern Highlands (n = 139), and Northern Piedmont (n = 133) (Figure 1). Sites in the validation data set represent a randomly selected subset of the larger data set and, for the most part, are distributed across the four ecoregions comparably to the development data set (Figure 1). To generate the best possible data set for model development, nested sites were removed but then used in the validation data set to provide the highest number and best distribution of sites for model validation.

For consistency, all watersheds were delineated for the selected sampling sites using USGS 7.5-minute quadrangle digital raster graphics (DRG) as base layers. The DRGs were overlayed with National Hydrography Data set (NHD) high resolution stream lines for each region [34]. Watershed boundaries were digitized at a scale of 1∶10,000 or larger. Adjacent watershed polygons were edge matched to eliminate all overlaps and gaps resulting in a consistent geospatial data set containing all study watersheds. Riparian buffer zone polygons were created within each watershed, extending 2 km upstream from the outlet of each watershed (i.e., point where the macroinvertebrate data was collected) along the main stem and all tributaries and 90 m on either side of the stream, similar to previous buffering approaches by Brenden et al. [35]. The selected reaches and associated buffer zone polygons were then saved as a new geospatial data set. To ensure that the riparian buffer zone polygons matched the watershed boundary polygons at the outlet, the buffered riparian polygons were overlayed on the watershed polygons and any areas extending beyond the existing watershed polygons were clipped and deleted. Landscape metrics derived from the spatial data sets were created for each watershed and riparian zone buffer and included ecoregion, elevation, slope, land cover (2001), road networks, soil infiltration capacity, hydrography, dams, pollution point sources, precipitation, population density, and canopy cover (Supplemental File: Appendix S1). All abbreviations for riparian based explanatory variables begin with the letters “Rip”; otherwise, variables are watershed based (Table 1).

### Hydrologic Runoff Metrics

Runoff is defined here as the total flow delivered to streams and rivers expressed on a per unit area basis. Runoff has the units of length divided by time and, when multiplied by upstream basin area, has the units of volume divided by time. The estimated runoff does not differentiate various sources of streamflow; it includes all sources of flow (groundwater discharge, rapid subsurface flow, and overland flow) delivered to streams (David Wolock pers. comm.), [36]. Estimates of hydrologic-unit runoff for all flow variables (Table 1) used for developing ecological models were derived using an area-weighted flow modeling approach that combines historical daily flow data collected at U.S. Geological Survey (USGS) streamgages, the respective drainage basin boundaries of the streamgaging stations, and the boundaries of the individual eight digit hydrologic cataloging units (HUC-8s) to develop a hydrologic unit runoff grid [36]. HUC-8 runoff values were used to estimate flow metrics for ungaged streams. This geospatial grid was used as the base layer to develop streamflow runoff estimates for the study basins. To derive these estimates, all 1058 of the previously delineated study basins were overlain onto the grid and run-off values in millimeters were computed. A total of 51 hydrologic runoff metrics were computed, which included the yearly average, minimum, and maximum runoff and coefficient of variation of runoff; the average, minimum, and maximum runoff for summer (Jul–Aug), winter (Jan–Feb), and spring (Apr–May); and the monthly average, minimum, and maximum for January–December. Refer to Brakebill et al. [36] for full description of how the hydrologic-unit runoff grid was developed for the coterminous U.S.

### Macroinvertebrate Data

Macroinvertebrate data from 1996 to 2009 assembled for this study were comparable in terms of sampling protocols (sampled habitat, number of composite samples, and total sampled area) and laboratory procedures, including sorting, subsample count level, and taxonomic resolution. In general, all macroinvertebrate samples were collected in wadeable riffle/run habitats using quantitative collection techniques (e.g., kick, Slack, or d-frame nets) from designated stream areas (typically five to eight separate collections combined to form a composite sample or a single running kick sample from a 5 m area) [37]–[42]. These data were extensively reviewed to ensure that the aggregated data included the same taxonomic groups, followed the same nomenclature, and had appropriate taxonomic resolution before data analysis was initiated. The Invertebrate Data Analysis System (IDAS) software [43] was used to resolve all taxonomic issues (taxonomic identification level and nomenclature, i.e., taxonomic harmonization), to remove ambiguous taxa [44] and to randomly subsample raw counts to an equal 100 specimen count (the highest possible subset for maintaining consistency across all ecoregions and programs). In general, data for dominant aquatic insect orders were resolved at genus level. Less common orders were often aggregated to family level. Rare organisms or those with difficult taxonomy were sometimes aggregated to order or higher. The dipteran family Chironomidae, for example, is considered an important bioindicator group, but historically a difficult group to identify to genus or species. Because of differences among the various state data sets, this group had to be aggregated to the family level. IDAS was used to generate approximately 120 macroinvertebrate richness and abundance metrics, including tolerance and functional group metrics, which were calculated using values from Barbour et al. [45]. A reduced set of metrics for analysis was obtained using a variety of exploratory analysis techniques including evaluating scatter plots for outliers, correlation of metrics to core watershed disturbance variables, correlation among macroinvertebrate metrics and results of previous analyses in different regions of the U.S. [7],[11],[22],[46]. From the reduced set, we selected four macroinvertebrate metrics as response variables for model development and validation: EPT Richness (richness of the orders Ephemeroptera, Plecoptera, and Trichoptera; EPTR), average tolerance of all taxa (RichTOL) based on supplemented EPA tolerance values, Richness of Intolerant Taxa (INTOL_RICH), and Non-Insect Richness (NonInsectR) (Table 1).

### Ethics

This paper uses only data already collected in previous studies; there are no ethical conflicts with animal use. All data were collected by either by the USGS or state agencies following appropriate state protocols for field sampling and permission access. No permits were required for sampling of macroinvertebrates and no protected species were sampled.

### Model Development

We developed BRT models for four macroinvertebrate metrics for the full region and for each of the four ecoregions separately. Regression trees fall within the commonly used classification and regression tree (CART) or decision tree family, and their use and technical details have been described (e.g., [23],[24],[25]) and extensively applied [5],[11],[17],[22],[27],[28],[47] in the literature; therefore, we will provide only a brief description. Boosted regression trees (BRT) are among a family of techniques used to advance single classification or regression trees by averaging the results for each binary split from numerous trees or forests, thus reducing the predictive error and improving overall performance [26],[27]. In BRT, after the initial tree has been generated, successive trees are grown on reweighted versions of the data, giving more weight to cases that are incorrectly classified than those that are correctly classified within each growth sequence. Thus, as more and more trees are grown in BRT, the large number of trees increases the chance that cases that are difficult to classify initially are correctly classified, thus representing an improvement to the basic averaging algorithm used in random forest [26]. Boosted trees and random forest models retain the positive aspects of single trees seen in CART models, yet have improved predictive performance, nonlinearities and interactions are easily assessed, and they can provide an ordered list of the importance of the explanatory variables [26],[47].

Although BRT offers improved modeling performance over CART, the simple single tree obtained from CART is lost, making it more difficult to visualize the results. Partial dependency plots (PDPs) provide a way to visualize the effect of a specific explanatory variable on the response variable after accounting for the average effects of all other explanatory variables [26],[27]; PDPs for selected variables important in models appear as examples in the results. BRT models were run using the gbm library in R and specific code from Elith et al. [27]. We used R^2^ and cross-validation R^2^ (CV R^2^) values to compare BRT models because they are well understood measures of the amount of variation explained by the models.

BRT models were developed using four macroinvertebrate response metrics (EPTR, RichTOL, INTOL_RICH, and NonInsectR) and a set of explanatory variables (watershed, riparian, hydrologic, and natural landscape; Table 1) that were evaluated using a variety of exploratory analysis techniques, including examining scatter plots for outliers and assessing intercorrelation among variables. In addition, all explanatory variables were screened and only those that had > 50% non-zero values were retained for further analysis. The BRT model was first developed using the model development data set. We used a bag fraction of 0.75, a learning rate of 0.001 to 0.0005 for developing our models, and a tree complexity of 5; a bag fraction of 0.75 means that each tree is developed using a random selection of 75% of the data. The learning rate influences the total number of trees evaluated for the model, while tree complexity controls whether interactions are fitted, a value of 5 allows the assessment of up to 5-way interactions. Variable relative importance (VRI) was calculated using formulae developed by Friedman [48] and implemented in the R gbm library to estimate the relative influence of predictor variables. Calculations of VRI are based on the number of times a variable is selected for splitting, weighted by the squared improvement to the models as a result of each split, averaged over all trees. The relative importance of each variable is scaled so that the sum adds to 100, with higher numbers indicating stronger influence on the modeled response. We developed our BRT models using a multi-stage process: (1) BRT models were first run with all watershed, riparian, and natural landscape variables only, with the top 10 variables in the variable relative importance list retained for further analysis, (2) BRT models were run with all hydrologic runoff based variables only, with the top 10 variables in the variable importance list retained for further analysis, and (3) BRT models were run combining the top 10 variables from steps 1 and 2. Finally, explanatory variables in the final BRT models were pruned by using a combination of VRI scores, evaluation of interactions and partial dependency responses and gbm simplify scores following the approach outlined by Elith et al. [27] to minimize overfitting. In brief, we deleted all variables with relative importance values less than 7. The remaining variables were then used to develop the final BRT model. The final model was selected by sequentially deleting variables and evaluating the effects on R^2^ and examining partial dependency plots; this was done independently for each invertebrate metric for each region. We then used the final BRT model to predict EPTR, RichTOL, INTOL_RICH, and NonInsectR values for the validation data set of 467 sites and calculate model performance measures. Finally, we regressed observed values against predicted values for both development and validation models as a visual measure of model precision and bias [49]. The sites in validation data set were not randomly selected from the full data set as would be ideal, but instead were extra nested sites that had been removed from the final full region data set. Nevertheless, the validation data encompassed a large number of sites equally distributed across all regions.

In order to get a large enough sample size, the data set analyzed had a large range in watershed size (8–780 km^2^) and mean elevation (23–870 m); so, in addition to the tests above regarding spatial scale, we wanted to determine whether breaking the data set into watershed and elevation classes would improve model performance. Stream size, that is, location along the continuum or gradient from the headwaters to the mouth, influences the type and distribution of the aquatic fauna [29]. Therefore, three watershed size and elevation classes were developed to evaluate whether improved model performance over the Full Region model could be achieved. We divided the Full Region model data set into three watershed size (8–27.0; >27.0<66.0; and >66.0<777.0 km^2^) and mean elevation classes (23–225, 225–450, and > 450 m). This division created relatively even sample sizes among the three classes, but also followed a recognized common regional break for small watershed sizes (± 26 km^2^). All BRT modeling was completed in R ([50], version 2.13.1).

## Results

Comparing within a region but across metrics, the average tolerance of all taxa (RichTOL) consistently had the highest R^2^ and CV R^2^ for BRT models among the four metrics for all the regions studied (Table 3). Given the use of the EPT Richness (EPTR) metric as a component of many multimetric indices in the northeast, it was unexpected that it would have the lowest R^2^ across all regions for BRT models when compared to the other metrics, except in the NE_High, where it had the second lowest value. The other two metrics, Richness of Intolerant Taxa (INTOL_RICH) and Non-insect Richness (NonInsectR), were, in general, intermediate in model R^2^ value to RichTOL and EPTR (Table 3).

**Table 3:** Comparison of model evaluation statistics for boosted regression tree models (BRT) for four macroinvertebrate metrics for development (develop) and validation (valid) data sets at two spatial scales (full region and four ecoregions), number of variables in final model in parentheses.

Metric	Model Data Set	Model Statistic	Full Region	North Central Appalachians	Ridge and Valley	Northeastern Highlands	Northern Piedmont
			n = 591	n = 167	n = 152	n = 139	n = 133
EPTR	Develop	R^2^	0.63 (6)	0.54 (4)	0.65 (5)	0.70 (4)	0.76 (4)
		CV R^2^	0.46	0.27	0.30	0.38	0.50
	Valid	R^2^	0.63	0.68	0.82	0.57	0.78
		CV R^2^	0.41	0.08	0.39	0.28	0.48
RichTOL	Develop	R^2^	0.77 (5)	0.67 (5)	0.81 (4)	0.80 (4)	0.84 (4)
		CV R^2^	0.64	0.42	0.51	0.56	0.65
	Valid	R^2^	0.73	0.64	0.64	0.68	0.82
		CV R^2^	0.59	0.23	0.34	0.44	0.62
INTOL_RICH	Develop	R^2^	0.66 (5)	0.60 (7)	0.65 (5)	0.67 (4)	0.72 (6)
		CV R^2^	0.49	0.27	0.33	0.43	0.42
	Valid	R^2^	0.66	0.70	0.67	0.59	0.70
		CV R^2^	0.48	0.26	0.42	0.30	0.39
NonInsectR	Develop	R^2^	0.68 (4)	0.66 (5)	0.69 (4)	0.75 (5)	0.72 (4)
		CV R^2^	0.56	0.34	0.49	0.43	0.48
	Valid	R^2^	0.67	0.31	0.80	0.70	0.80
		CV R^2^	0.53	0.02	0.57	0.39	0.62

Validation models run with the same variables as the final development model.*

*R^2^–adjusted R-squared, CV R^2^-Cross validation R^2^; EPTR–Total taxa richness of Ephemeroptera (mayflies), Plecoptera (stoneflies) and Trichoptera (caddisflies); RichTOL-Average tolerance of all taxa; INTOL_RICH-Richness of intolerant taxa; NonInsectR-Noninsect taxa richness.

Spatial scale results showed that relatively strong explanatory models with R^2^ values ranging from 0.63 to 0.77 were developed using BRT techniques for the Full Region models (Table 3). In all cases, however, the N_Pied produced stronger models and the NC_App produced weaker models than those produced in the Full Region models. In the other regions (R&V and NE_High) the results were mixed and in some cases the Individual Ecoregion models explained slightly more variation than the Full Region models. The cross-validation R^2^ values, which are much more conservative than adjusted R^2^, decreased significantly across all metrics and regions compared to R^2^, the decrease ranging from 12 to 33 points per model (Table 3). NC_App, which had the smallest range or gradient in land use disturbance (e.g., urbanization, manmade channels, dam density, and agricultural land use) had the lowest R^2^ for each metric across the regions and the largest average decrease in CV R^2^.

Across the four metrics, the final Full Region BRT models had between four and six explanatory variables (Table 4): a variable related to urbanization (population density, percent urban, or percent manmade channels), a measure of hydrologic runoff (average December or maximum monthly runoff) and a natural landscape variable (slope and elevation) were in every model. Other explanatory variables in the Full Region models were percent riparian forest, riparian canopy, and percent forest in the watershed. Models developed across the four ecoregions showed the same pattern related to urbanization as the Full Region models. Every model had at least one, and in some cases, more than one, urbanization-related variable. Similar to the Full Region models, many of the Individual Ecoregion BRT models had forest or riparian canopy-related variables and over half of the models had some form of natural landscape factors. In addition, 9 of the 16 Individual Ecoregion models (56.2%) had a hydrologic runoff variable, and in some cases 2 hydrologic variables (e.g., the NonInsectR models for R&V and NE_High). In six out of the nine (66%) cases where a hydrologic variable entered the BRT model for the Individual Ecoregions, it was ranked either first or second in importance. Urbanization-related, riparian forest, and hydrologic runoff variables were consistently selected as the top variable with almost equal frequency across all 20 BRT models; however, only in one (the RichTOL model for N_Pied) was a natural landscape factor selected first (Table 4).

**Table 4:** Comparison of explanatory variables for boosted regression trees (BRT) models for four macroinvertebrate metrics at two spatial scales (Full Region and four Individual Ecoregions); variables are presented in descending order of variable relative importance (VRI) in each model.*

Invertebrate Metrics	Full Region		North Central Appalachians		Ridge and Valley		Northeastern Highlands		Northern Piedmont	
	n = 591		n = 167		n = 152		n = 139		n = 133	
		VRI		VRI		VRI		VRI		VRI
EPT Richness	Urban	34	Min_April	29	Rip.Forest	32	Urban	51	Urban	46
(EPTR)	Rip.Slope	19	Rip.Pop.Density	27	Ag+Urban	21	Wetland	17	Mean Elevation	26
	Ave_Dec	14	Rip.MeanCanopy	25	Pop.Density	18	ManMadeChan	16	Rip.Forest	18
	Rip.MeanCanopy	12	Road Density	20	Max_MonthCV	17	Rip.Forest	16	ManMadeChan	10
	Rip.Forest	12			Urban	12				
	ManMadeChan	9								
Average	Pop.Density	31	Rip.Forest	36	ManMadeChan	33	Ave_March	48	Mean Elevation	51
Tolerance of all	ManMadeChan	25	Rip.Pop.Density	17	Rip.Forest	29	Rip.Wetland	27	ManMadeChan	22
Taxa	Rip.Slope	16	Rip.Ag	17	Max_Nov	20	Urban	15	Urban	15
(RichTOL)	Rip.Forest	14	Pop.Density	16	Pop.Density	18	Road Density	10	Rip.Forest	12
	Ave_Dec	14	Soil Infiltration D	14						
Richness of	Rip.Forest	34	Rip.Forest	22	Rip.Forest	31	Urban	43	Urban	28
Intolerant Taxa	Pop.Density	23	Rip.Slope	18	Pop.Density	24	Ave_July	25	Rip.Max.Elev	20
(INTOL_RICH)	Forest	17	Soil Infiltration D	14	Urban	16	Rip.Forest	19	Mean Slope	18
	Rip.Max.Elev	17	Min_April	13	Rip.MeanCanopy	16	ManMadeChan	14	Soil Infiltration B	14
	Max_Monthly	10	Rip.Ag	13	Mean Slope	13			Rip.Wetland	11
			Rip.Pop.Density	12					Pop.Density	10
			Road Density	9						
Noninsect	Ave_March	41	Rip.Forest	25	Max_March	44	Max_January	24	Max_April	36
Richness	ManMadeChan	26	Pop.Density	24	Max_May	24	Ave_March	24	Rip.Max.Elev	27
(NonInsectR)	Mean Elevation	17	ManMadeChan	22	Soil Infiltration C	21	Rip.Wetland	23	Soil Infiltration C	20
	Rip.Slope	16	Dam Density	15	ManMadeChan	11	Rip.Slope	18	ManMadeChan	17
			Rip.Pop.Density	14			Pop.Density	12		

*EPTR –Total taxa richness of Ephemeroptera, Plecoptera and Trichoptera; B–Soils with a moderate infiltration rate, C–Soils with a slow infiltration rate, D–Soils with a very slow infiltration rate, Rip –Riparian. See Table 1 for variable definitions.

**Figure 2:**
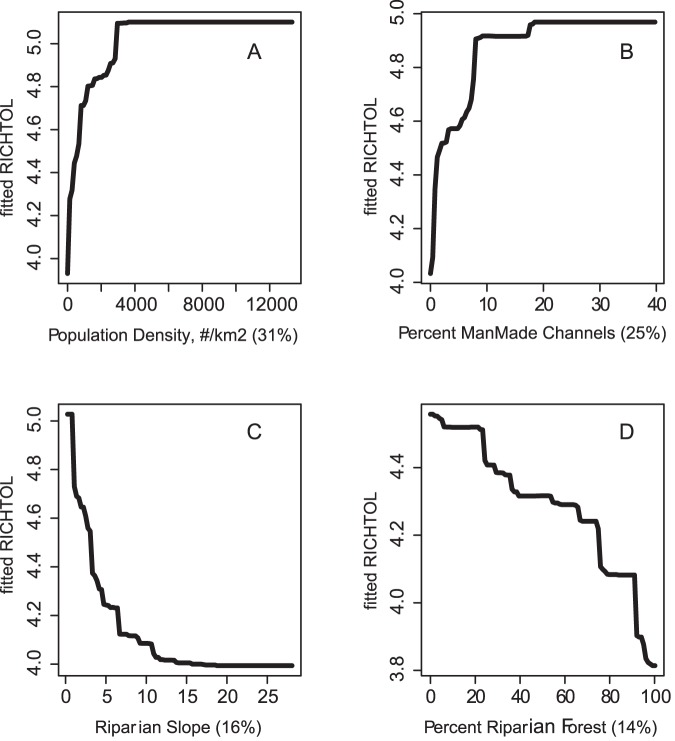
Partial dependency plots for variables in BRT model for RichTOL for the Full Region. Boosted regression tree partial dependency plots show the response form of average taxa tolerance (y-axis  =  fitted function of RichTOL) based on the effect of individual explanatory variables with the response of all other variables removed (development data set). Shown in order of model importance: (A) population density (numbers/km^2^), (B) percent manmade channels, (C) riparian slope and (D) percent riparian forest, model R^2^ = 0.77. The relative contribution of each explanatory variable is reported in parentheses. Refer to Table 1 for variable definitions. The top two variables for the Full Region model showed potential threshold type responses for urbanization variables and the third variable, a natural factor, is likely acting as an urban surrogate (riparian slope). The final variable, a measure of riparian disturbance follows a more linear response and along with urbanization variables was a common explanatory variable in most models among the different regions.

**Figure 3:**
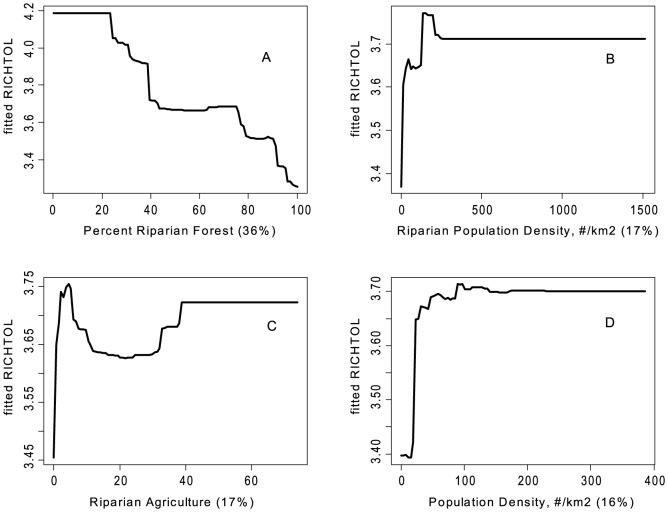
Partial dependency plots for variables in BRT model for RichTOL for North Central Appalachian Region. Boosted regression tree partial dependency plots show the response form of average taxa tolerance (y-axis  =  fitted function of RichTOL) based on the effect of individual explanatory variables with the response of all other variables removed (development data set). Shown in order of model importance: (A) percent riparian forest, (B) riparian population density (#/km^2^), (C) percent riparian agriculture and (D) population density (#/km^2^). The relative contribution of each explanatory variable is reported in parentheses. Refer to Table 1 for variable definitions. Three of the four variables can be interpreted as disturbance variables, two directly assessing urban land use (population density) and the third, riparian forest, which measures the amount of disturbance in the riparian zone was the top variable modeled. However, this region had the shortest disturbance gradient and the lowest modeled R^2^ (0.67), though still relatively strong.

**Figure 4:**
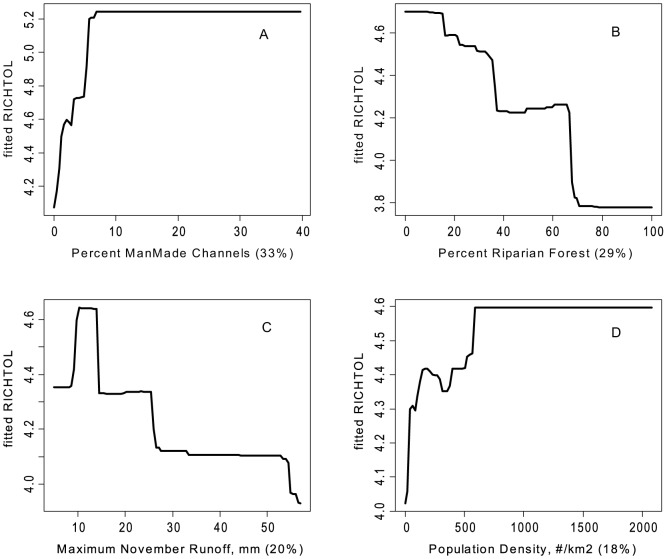
Partial dependency plots for variables in BRT model for RichTOL for Ridge and Valley Region. Boosted regression tree partial dependency plots show the response form of average taxa tolerance (y-axis  =  fitted function of RichTOL) based on the effect of individual explanatory variables with the response of all other variables removed (development data set). Shown in order of model importance: (A) percent manmade channels, (B) percent riparian forests, (C) maximum November runoff (mm) and (D) population density (#/km^2^), model R^2^ = 0.81. The relative contribution of each explanatory variable is reported in parentheses. Refer to Table 1 for variable definitions. Three of the four variables measure the effects of disturbance, two measure the response to urban land use and the other disturbance in the riparian zone due to either agriculture or urbanization. The fourth variable shows the response due to maximum November runoff.

**Figure 5:**
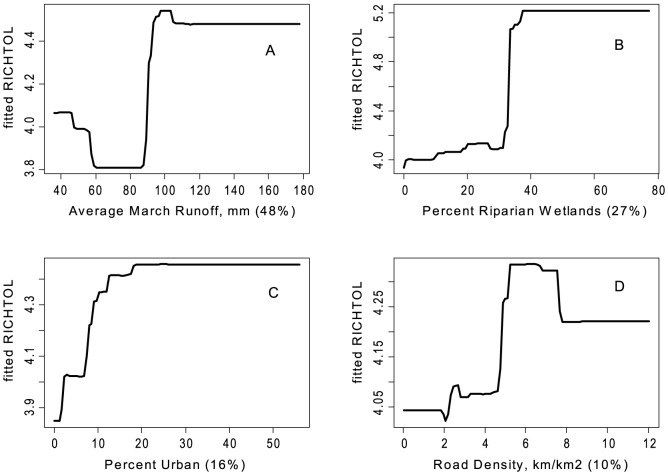
Partial dependency plots for variables in BRT model for RichTOL for NE Highlands Region. Boosted regression tree partial dependency plots show the response form of average taxa tolerance (y-axis  =  fitted function of RichTOL) based on the effect of individual explanatory variables with the response of all other variables removed (development data set). Shown in order of model importance: (A) average March runoff (mm), (B) percent riparian wetlands, (C) percent urban and (D) road density (km/km^2^), model R^2^ = 0.80. The relative contribution of each explanatory variable is reported in parentheses. Refer to Table 1 for variable definitions. All four explanatory variables modeled can be interpreted as an urbanization land use effect. Average March is expected to increase due to higher imperviousness with higher urbanization and percent riparian wetlands we believe is acting as a surrogate for urbanization; higher wetlands commonly occur in lower elevation valleys where there is commonly more urban development. The last two variables measure urban land use directly.

**Figure 6:**
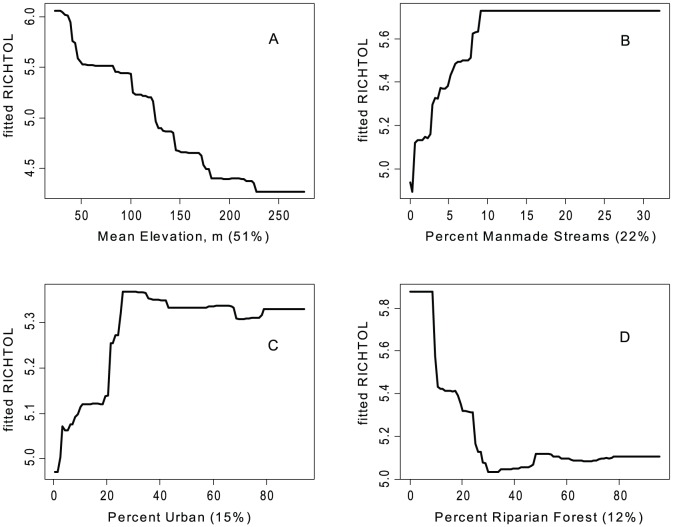
Partial dependency plots for variables in BRT model for RichTOL for Northern Piedmont Region. Boosted regression tree partial dependency plots show the response form of average taxa tolerance (y-axis  =  fitted function of RichTOL) based on the effect of individual explanatory variables with the response of all other variables removed (development data set). Shown in order of model importance: (A) mean elevation (m), (B) percent manmade channels, (C) percent urban and (D) percent riparian forest, model R^2^ = 0.84. The relative contribution of each explanatory variable is reported in parentheses. Refer to Table 1 for variable definitions. This was the only model that had a natural factor as the top explanatory variable, however, we believe elevation is acting as a strong surrogate in this region for urbanization, though it is likely more complex than a strictly one for one surrogate. The other three variables all measure directly or indirectly urban land use disturbance and show a relatively strong potential threshold type response.

**Figure 7:**
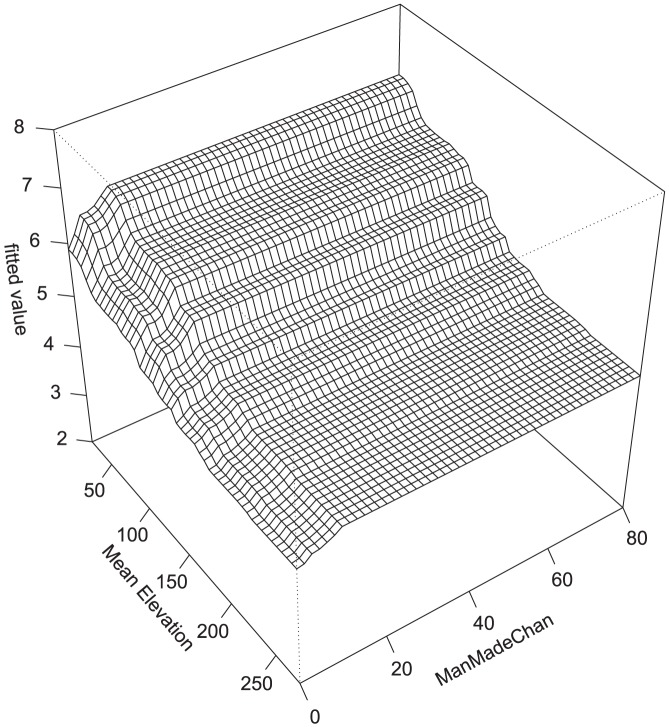
Interaction of manmade streams and mean elevation on RichTOL for Northern Piedmont BRT model. Boosted regression tree partial dependency plot shows the response form of average taxa tolerance (y-axis  =  fitted function of RichTOL) based on the effect of the interaction of two individual explanatory variables along the response variable (all other variable responses removed). There is a relatively strong interaction acting on RichTOL at low values of mean elevation and high values of percent manmade streams that cause high values of tolerant taxa to occur. This is a common pattern, higher urbanization occurring in the lower elevation valleys.

**Figure 8:**
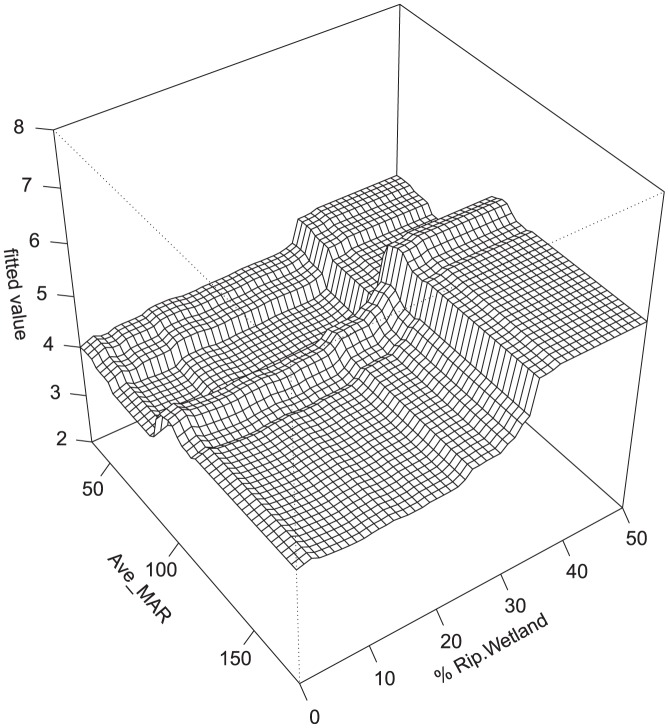
Interaction of riparian wetlands and average March runoff (mm) on RichTOL for N.E Highlands BRT model. Boosted regression tree partial dependency plot shows the response form of average taxa tolerance (y-axis  =  fitted function of RichTOL) based on the effect of the interaction of two individual explanatory variables along the response variable (all other variable responses removed). There is a relatively large interaction at high values of average March runoff when there are also high values of percent riparian wetland thus resulting in higher values of tolerant taxa (RichTOL) than would be expected. We believe that high values of riparian wetland are acting as a surrogate for high values of percent urban land use.

There were some common response patterns in the urbanization-related explanatory variables related to RichTOL. For example, the partial dependency plots for population density, road density, percent manmade channels, and percent urban showed a relatively abrupt threshold-type response (Figs. 2A,B; 3B,D; 4A,D; 5C,D; and 6B,C) (Note: to reduce complexity, only the top four variables are shown in each partial dependency plot). However, response patterns for percent riparian forest were mixed and showed a more linear response for the Full Region (Figure 2D) as compared to the stepped response and strong threshold seen for the NC_App and R&V and N_Pied regions, respectively (Figs. 3A, 4B, and 6D). The responses for hydrologic runoff variables also tended to show a threshold or stepped type response (Figure 4C and 5A, respectively). There were also some clear interactions among important explanatory variables, including natural landscape factors and disturbance variables. For example, at low elevations in the N_Pied region there are a range of values of manmade channels, with high values (threshold at about 15% manmade channels) resulting in high values of tolerant taxa (RichTOL), but at high elevations there are only low values of manmade channels, resulting in low values of RichTOL (Figure 7). As another example, the combination of high values of average March runoff and high values of riparian wetlands interact in the result of the highest values of RichTOL. At low values of March runoff there are relatively low values of RichTOL even across a range of values for riparian wetlands, except at the highest wetland values, yet these values are not as high as when there is high March runoff in combination with high wetland values (Figure 8). For brevity, the response form as shown in the partial dependency plots for the other invertebrate metrics for each region are not shown here but provided as supplemental material online (Figure S1).

**Figure 9:**
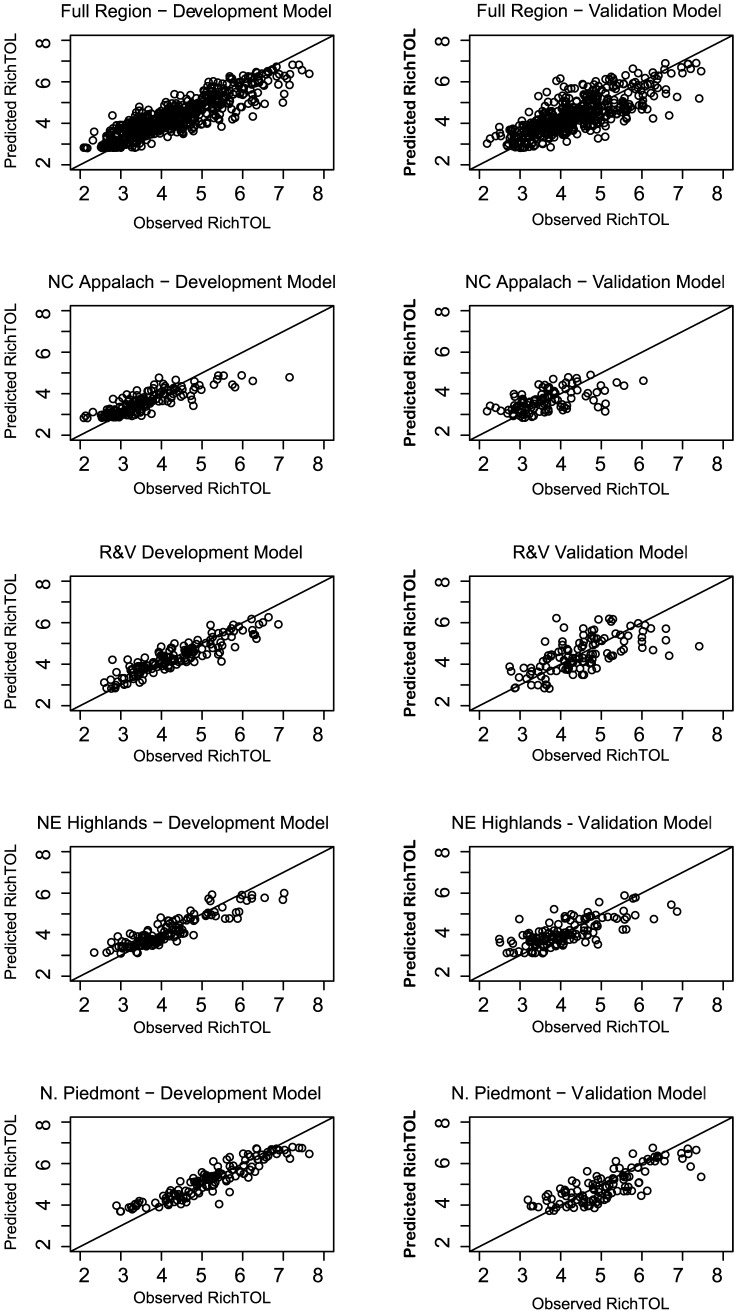
Observed versus predicted plots for BRT models for development (left) and validation (right) data sets. The observed versus predicted plots are based on the boosted regression models developed for average taxa tolerance (RichTOL) for five models: Full Region and four individual ecoregions (NC Appalachian, Ridge and Valley, NE Highlands, and N. Piedmont). The Full Region and N. Piedmont region plot relatively tight to the 1∶1 line for both the development and validation models indicating a good predictive fit with only slight bias at high and low values of RichTOL. The other regions in general showed more scatter and the N.C. Appalachian region which had the lowest modeled R^2^, had had the shortest disturbance gradient (narrow range of RichTOL values) compared to the other regions.

Validation model results varied across metrics and regions, 10 out of the 20 total models had R^2^ values for the validation models within five points of the development models. Of the remaining 10 models, five had validation R^2^ values lower than the development models (range −8 to −35) and five had higher values (range +8 to + 17). Models with higher performance for the development data set tended to also have higher R^2^ values in the validation data, the exception were those models where the validation values were higher. Observed vs. predicted plots (Figure 9) are shown only for RichTOL, primarily because it consistently represented stronger BRT models with greater predictive power (highest R^2^ values) than the other metrics assessed in this study and to avoid adding unnecessary length to the manuscript. Observed vs. predicted plots for all other metrics and regions are provided as supplemental material online (Figure S2). In general, the observed-predicted plots reveal that for all metrics and regions, the models over predict at low values and under predict at high values for each invertebrate metric, the extent of the bias varied with the R^2^ of the models. RichTOL for the Full Region models represented the largest range in the disturbance gradient and reflected only a slight under prediction bias in the upper end of the values for the development model (n = 591; Figure 9 left panel) as compared to wider and more even distribution of points across the full range of values for the validation model (n = 467; Figure 9 right panel). As would be expected, the Individual Ecoregion models, which have an overall smaller sample size, reflected a shorter disturbance gradient that varied across regions. The NC_App ecoregion encapsulated the shortest gradient (range in RichTOL values; Figure 9) and reflected a distinct bias for the development and validation models, under and over predicting tolerance values near the high and low end of its range, respectively (n = 167). R&V and NE_High covered a slightly longer portion of the disturbance gradient than NC_App with values more tightly clustered along the 1∶1 line for the development models, yet also less tightly clustered around the 1∶1 line for the validation models (Figure 9). The N_Pied region which had the strongest development model encapsulated a relatively long portion of the disturbance gradient and reflected only slight bias at low and high tolerance values. However, the validation model for N_Pied showed slightly more scatter around the 1∶1 line compared to the development model, although still less bias than all the other regional validation models (Figure 9).

BRT models developed for watershed size classes for the same metrics tended to show similar patterns in response as the ecoregion models. For example, RichTOL had the highest overall model performance of the four metrics across all watershed size classes and EPTR had the lowest performance (Table 5). There was little or no overall improvement in model performance for the majority of the watershed size classes compared to the Full Region models. The largest improvements in R^2^ occurred for RichTOL (watershed size class 2), NonInsectR (watershed size class 1), and EPTR (watershed size class 2) which slightly exceeded the Full Region model results by 0.06, 0.09, and 0.06, respectively (Table 5). We also tested model performance for three classes of elevation (mean elevation: 23–225, 225–450, and > 450 m) and similar to the result for models for watershed size classes, found little or no improvement compared to the Full Region models (for brevity, data not shown).

**Table 5:** Comparison of model evaluation statistics for four macroinvertebrate metrics for three watershed size classes, number of variables in final model in parentheses.*

Macroinvertebrates	Model Type	Model Statistic	Full Region	WS Size Class 1 (8 – 27 km^2^)	WS Size Class 2 (>27<66 km^2^)	WS Size Class 3 (> 66<777 km^2^)
			n = 591	n = 282	n = 188	n = 121
EPT Richness	BRT	R^2^	0.63 (6)	0.64 (5)	0.69 (4)	0.65 (4)
(EPTR)		RMSE	2.34	2.68	2.30	2.18
Average Tolerance of	BRT	R^2^	0.77 (5)	0.78 (4)	0.83 (4)	0.68 (4)
all Taxa (RichTOL)		RMSE	0.53	0.58	0.46	0.39
Richness of Intolerant	BRT	R^2^	0.66 (5)	0.68 (4)	0.67 (4)	0.63 (4)
Taxa (INTOL_RICH)		RMSE	2.36	2.55	2.12	2.09
Noninsect Richness	BRT	R^2^	0.68 (4)	0.77 (4)	0.73 (4)	0.72 (4)
(NonInsectR)		RMSE	1.39	1.28	1.25	2.09

*BRT – Boosted Regression Trees. R^2^–adjusted R-squared, CV R^2^—cross-validation R^2^, EPTR–Total taxa richness of Ephemeroptera, Plecoptera and Trichoptera.

## Discussion

The primary objective of this study was to evaluate the influence of scale (Full Region models versus four Individual Ecoregion models) on model performance by developing predictive benthic-macroinvertebrate boosted regression tree (BRT) response models using readily delineated and commonly applied watershed variables. Our working hypothesis was that small scale Individual Ecoregion models would outperform larger, Full Region models. Previous research has shown that large scale assessments, in general, do not perform as well as smaller local or regional scale models largely due to inherent differences in biogeography and disturbance type or extent [5],[6],[12],[14],[15],[16]. Waite [51] and Riseng et al. [52] also found that the strength of correlations of macroinvertebrate and algal metrics to agricultural disturbance variables (agricultural intensity and nutrient concentrations), varied with spatial scale and geographic region. Based on this weight of evidence, we hypothesized that the larger scale Full Region models would be more generic, have lower predictive power, and would likely include more natural landscape variables (e.g., slope and elevation, etc.) as compared to the smaller scale Individual Ecoregion models. Our modeling results, however, did not support this hypothesis. For example, the Full Region BRT models were, in some cases, as strong or stronger than the Individual Ecoregion models (e.g., they were consistently stronger than all the North Central Appalachian models; Table 3), and land use, hydrologic runoff, and other disturbance related variables were frequently important explanatory variables in these models (Table 4). The Full Region models, with the exception of NonInsectR, only had one natural landscape variable out of the four to six variables in each of the final models. Some Individual Ecoregion BRT models did show significant improvement in model performance (e.g., gains ranged from a minimum in R^2^ of 0.01 for the Northeastern Highlands to a maximum of 0.13 for Northern Piedmont); however, natural landscape variables were equally as common in the Individual Ecoregion models as they were in the Full Region models. One possible explanation for why these findings tended not to coincide with our original hypothesis may be that the Individual Ecoregions used in this study did not have as strong a biogeographic gradient when compared to regions modeled in other recent studies (e.g., Western U.S. in [14],[17]) or perhaps the larger sample size used to develop the Full Region models resulted in longer and more defined disturbance gradients for model development. In either case, our results may indicate that models of broader spatial extent, such as the Full Region models evaluated in this study, could represent an important starting point for prediction of changes in macroinvertebrate response at unsampled stream sites. These results tend to support the need to develop models at the larger regional scales, but also indicate that some improvement in model performance may be provided by smaller scale efforts using Individual Ecoregions or other common surrogates (e.g., physiographic provinces). The extent of improvement, however, may directly depend upon the extent of the gradient encapsulated by the data set relative to the scale of the investigation and in the synchronicity in scales between the biota and the landscape (i.e., are the biota responding at one scale while landscape data and related variability occurs at another scale).

Even though the validation data set was not ideal since it was created from “left over” nested sites and not sites randomly subset from a larger pool of sites, it still provides a measure of how well the models are likely able to predict at unsampled sites. Fifteen out of the 20 validation models had R^2^ values that were no lower than five points less than the development models or had values that were actually higher. The Full Region and N_Pied region still had some of the higher R^2^ values for the validation data than the other regions but there was more variability among the metrics within a region and among regions as to which had the higher performing validation models. Looking at the Observed vs Predicted plots for the development and validation models, it becomes clear that even the strongest models have bias at the low and high ends of the metric ranges and that the validation data had a slightly different range in observed values than the development data (Figure 9 and supplemental material). For example, the models frequently would over predict richness values at low values (three metrics were based on richness), rarely predicting zero or one's even though these were observed in the two data sets (seen as values above the 1∶1 line in plots). The opposite occurred at higher values, the models often under predicted at the higher ranges (seen as values below the 1∶1 line in plots). As would be expected, this bias was directly related to measures of model performance, models with higher R^2^ had less low and high bias than those models with lower R^2^ values. However, a lot could be learned by investigating why some sites were predicted to have poor or higher invertebrate values than were observed. Since the models primarily use land use at the watershed and riparian scales, perhaps these sites were better or worse than expected in water quality or habitat condition than most sites with similar land use condition. Across all models, the more conservative CV R^2^ showed significant decrease in values compared to the standard adjusted R^2^ model performance measure, suggesting that there may be some level of over-fitting in the models.

Across all the models, two clear patterns emerged: (1) some measure of urbanization, the amount of riparian disturbance (e.g., percent riparian forest, riparian population density), and hydrologic runoff were important explanatory variables, and (2) many of the explanatory variables showed a distinct threshold-type response (shown in the PDPs as transition points in ecological condition [53]–[55]). For example, a threshold response was commonly seen for the metric RichTOL as predicted by population density, percent urbanization, and percent manmade channels (Figs. 2A, 5C, and 6B, respectively). Note that these are only potential thresholds; more analysis and evaluation would have to be conducted to determine if there are actual thresholds in this data [55]. Land use in parts of the Northeast tends to be dominated by urbanization rather than agriculture, so it is not unexpected that variables representing urbanization or urban-related disturbance processes (e.g., population density and road density) were dominant factors in many of the predictive models developed in this study. The effects of urbanization have been well documented by other researchers ([16],[18],[19],[56]–[61], and many more); therefore, that topic will not be addressed here. The amount of stream channel alteration characterized in this study by the percentage of manmade channels, however, is seldom discussed in the urbanization literature, yet it was one of the variables commonly modeled in this study and represents an important anthropogenic pathway affecting stream communities that needs to be more broadly considered in future ecological modeling studies. In addition, more research is emerging on the importance of riparian integrity related to stream condition (e.g., [17],[51],[52],[62]) thus revealing riparian's critical influence on stream integrity. For example, Clapcott et al. [5] recently found that the amount of native riparian vegetation was the dominant variable in BRT models describing macroinvertebrate metrics in New Zealand and that riparian and urbanization variables showed threshold responses, similar to the responses seen for urbanization-related and riparian variables in this study. However, variables like population and road density and percent manmade channels, which are frequently highly skewed, are notoriously difficult to analyze via linear parametric techniques like MLR and this difference may be another reason why BRT models generally outperform MLR models.

The average tolerance of all the taxa in a sample (RichTOL) generally had the best model performance (highest R^2^) across all regions when compared to the other metrics. The richness of Ephemeroptera, Plecoptera, and Trichoptera taxa (EPTR), a common component of multimetric indices in the northeastern U.S., on the other hand, generally had the worst performance across all regions when compared to the other metrics. This is in contrast to what we found in development of models in the western U.S., where some measure of EPT richness and RichTOL were the two best response variables of all the macroinvertebrate metrics tested [11]. Most water resource agencies in the U.S. use a multimetric approach to evaluate the effect of anthropogenic stressors on aquatic systems [45], primarily because multimetric indices (MMIs) incorporate information from a number of metrics to provide a meaningful measure of overall biological condition. In addition, most state agencies that develop MMIs use some form of EPT as a component metric (e.g., percent EPT or EPT richness). Our finding that RichTOL was the most predictive metric is interesting (and to some degree confounding) because it may indicate that RichTOL could potentially be a highly useful component of state MMIs or BIBIs, especially given its strong predictive capability across all northeastern ecoregions studied. RichTOL, although sometimes considered in MMI development, is seldom used as a component of MMIs, more often a measure of total taxa richness or Hilsenhoff's Biotic Index [63] is used instead (e.g., New Jersey Impairment Score [40] and Pennsylvania Index of Biotic Integrity [64]). Bioassessment programs are continuously trying to improve criteria in support of MMI development and BRT models that explicitly show the utility of metrics such as RichTOL for predicting ecological changes along a disturbance gradient further support such efforts.

Because many of the variables used in the analysis did not follow a linear response form (see Figures 2–6), they are not likely to be identified as significant in multiple linear regression models, even when transformed. Therefore, it is possible that since MLR models assume linearity that they may sometimes underestimate the explanatory power of nonlinear relations in some variables. BRT is more robust and allows the inclusion of more variables in the model building phase than MLR, which permits easier testing for interaction effects and produces a list of variables explaining the importance of variation in the response variable. In addition, partial dependency plots from BRT can offer valuable insight into the pattern or form of the response based on select explanatory variables, thus improving model interpretation. Although BRT models have only recently begun to be applied in ecology, a number of researchers have shown the strength and promise of this modeling technique compared to other methods [5],[17],[26]–[28],[46]. It is evident from the results of this and other studies that BRT represents a more powerful modeling technique than MLR (and many other methods) for evaluating the effects of human alteration of the environment on aquatic communities.

For this study, we were also interested in determining whether predictive models could be improved by breaking the sites up into three distinct watershed size classes. Recent studies have shown that human disturbance variables (i.e., land use and water quality) in combination with natural landscape factors such as climate, stream size, and elevation may help account for the greatest amount of variation in biotic indicators [5],[11],[46],[65]. Thus, to broaden our understanding of scale effects on macroinvertebrate response model development and prediction, we felt it important to take factors such as stream size into account as it is well established that processes along the river continuum, including variation in landscape and riparian disturbance, water quality, habitat, and streamflow, affect the distribution and abundance of macroinvertebrates ([29] and numerous other authors). However, BRT models developed for the individual watershed size classes did not greatly improve overall model performance when compared to the Full Region model results (Table 5). Two plausible explanations for this are (1) the range in watershed size classes (8.0–777.0 km^2^) were not large enough to elicit a significant response and (2) changes along the stream size gradient tended to follow changes in landscape disturbance and elevation. Although stream size range was not extreme, it was large enough that we anticipated we would see an improved response; however, human landscape disturbance often follows an elevation gradient that may be acting as a surrogate for stream size. That is, smaller headwater streams tend to be in higher gradient areas (foothills and mountains) and larger streams are often in low elevation valleys [10],[11],[66]. However, we also tested model performance for three classes of elevation (mean elevation: 23–225, 225–450, and > 450 m) and similar to the result for models for watershed size classes, found little or no improvement compared to the Full Region models (data not shown). On its own, the range in elevation in this study did not seem to affect model performance; nevertheless, we believe there was interaction between elevation and land use variables that was important. This was exemplified in the PDPs, where there was interaction between elevation (and by extension, stream size) and percent man-made channels, a surrogate for urbanization. This interaction between elevation or other natural landscape variables and land use variables, which was common in this study, has also been shown in many other recent studies [6],[10],[11],[19],[22]. In essence, the greater predictability anticipated by developing models based on watershed size classes, was largely obscured by the interaction between watershed elevation and human disturbance. In the Northern Piedmont, elevation and urbanization closely follow each other, so even though there is not a large range in elevation in this region, elevation was the most important variable because it also expresses the effects of urbanization.

Stream hydrology, as represented by the proportion of hydrologic runoff, was an integral component of many of the predictive BRT models developed for this study (Table 4). The relation between aquatic community response and changes in hydrologic processes is well established. Poff et al. [67] emphasized the fundamental importance of inter- and intra-annual hydrologic variation as the primary controlling factor for sustaining the ecological integrity of streams, and nearly two decades of hydroecological research (see review by [68]) have substantiated this connection. Previous predictive modeling studies in the western U.S. using BRT (e.g., [11],[17],[22]) did not include hydrologic variables in their models predicting macroinvertebrate metrics or BIBIs, respectively, due to a dearth of hydrologic data availability across the region of study. Although speculative, it may have been possible to improve model performance in these studies by the inclusion of hydrologic attributes, especially hydrologic attributes that are commonly altered by anthropogenic processes. Many of the hydrologic runoff variables identified in this study were an integral part of the models developed and were strong predictors of macroinvertebrate-assemblage composition. For example, 75.0% of the Full Region and 56.3% Individual Ecoregion models developed included a hydrologic runoff variable with a high degree of predictive power and high relative variable importance (Table 4). These findings are highly consistent with the results of other studies that point to changes in annual streamflow processes as being a significant driver of changes in assemblage structure and function (e.g., [7],[69]–[75]. In some cases, hydrologic runoff was the most important variable (e.g., Average March and Maximum April for the NE_High and N_Pied, respectively) and notably, many of these hydrologic runoff variables represent periods of annual streamflow variability that are important to the timing of macroinvertebrate emergence and reproduction (spring high and low flows; maximum and minimum April, average March, and maximum May runoff; Table 4). Changes in assemblage structure due to modified annual flow patterns have been commonly associated with a disruption or alteration of life-history or behavioral cues [76]–[78]. For example, emergence periods for more sensitive taxa (taxa with less plastic life histories) like Ephemeroptera, Plecoptera, and some Trichoptera may be affected by alterations in mean annual spring runoff. Therefore, it is no surprise that in three of five EPTR and four of five NoninsectR predictive models (Table 4), hydrologic runoff was of high relative importance and in a number of cases was either the first or second most important variable. This finding further emphasizes the importance of developing models that include streamflow attributes, which encapsulate variability across the full hydrologic regime, especially for parts of the hydroperiod that are critical to the health and survival of aquatic communities.

### Implications

Even though spatial scale of the Full Region modeling area in this study was not extensive (i.e., it did not encapsulate the entire northeastern U.S.), it did include parts of four eastern U.S. states and contained four proximal ecoregions. In contrast to our initial hypothesis, however, we found that the Full Region models did almost as well in predicting macroinvertebrate structure as the more area specific Individual Ecoregion models. It may, therefore, be valuable for large regional models to be developed as a good preliminary assessment of major factors affecting the biological condition of streams in a geographic region as long as the range in natural landscape variability (e.g., climate, slope, elevation) can be minimized. The regional models can then be tested and compared against smaller subregional or watershed models to determine whether there is a large enough improvement in model performance or variable specificity to justify the general application of the regional models or more specificity is needed from local models.

There is an obvious trade-off in development time and model specificity between the Full Region and Individual Ecoregion models. In some instances, the Full Region model may be sufficient for understanding broader implications of human-derived disturbance; however; smaller subregional or local models that provide more detailed information on cause-effect processes (e.g., exemplify the utility of specific metrics such as RichTOL) are generally more advantageous for bioassessment programs and may ultimately be more useful from a management or regulatory perspective. We cannot emphasize enough the importance and critical nature of riparian vegetation related to stream integrity revealed in this and other studies. Although our models were relatively successful in predicting macroinvertebrate metrics based on land use and hydrologic runoff, there may be additional explanatory variables that we did not use that could improve model prediction (other landscape data, instream habitat, water chemistry, etc.). These additional variables would not typically be available for all unsampled streams, where one of the benefits of the models developed in this study is that they can be used to develop maps of macroinvertebrate metric response at unsampled stream reaches while including some measure of error. This last point is where future modeling assessments could benefit both the science and management communities: development and testing of models predicting biological condition at unsampled streams under various scenarios. If possible, models should be tested on independent validation data sets to assess potential over-fitting and model bias.

## Supporting Information

Figure S1 **Partial dependency plots for variables in BRT model for the other three invertebrate metrics for all the Regions.** Boosted regression tree partial dependency plots show the response form for invertebrate metrics (y-axis  =  fitted function of invertebrate metric) based on the effect of individual explanatory variables with the response of all other variables removed (development data set); variables shown in order of model importance. The relative contribution of each explanatory variable is reported in parentheses. Refer to Table 1 for variable definitions.
(DOCX)

Figure S2 **Observed versus predicted plots for BRT models for development (left) and validation (right) data sets for the other three invertebrate metrics.** The observed versus predicted plots are based on individual boosted regression models developed separately for three invertebrate metrics for all five models: Full Region and four individual ecoregions (NC Appalachian, Ridge and Valley, NE Highlands, and N. Piedmont).
(DOCX)

Appendix S1 **List of GIS data sets and their sources.**
(DOCX)
