# Supplementary material for: Stream Macroinvertebrate Response Models for Bioassessment Metrics: Addressing the Issue of Spatial Scale
Source: PLoS One. 2014 Mar 27;9(3):e90944. doi: 10.1371/journal.pone.0090944 (PMC3968005; doi:10.1371/journal.pone.0090944)
Supplement: Figure S1 — Partial dependency plots for variables in BRT model for the other three invertebrate metrics for all the Regions. Boosted regression tree partial dependency plots show the response form for invertebrate metrics (y-axis = fitted function of invertebrate metric) based on the effect of individual explanatory variables with the response of all other variables removed (development data set); variables shown in order of model importance. The relative contribution of each explanatory variable is reported in parentheses. Refer to Table 1 for variable definitions. (DOCX) [file pone.0090944.s001.docx]

**PlosOne Supplemental Material Description (Waite et al. 2014): This file contains the Partial Dependency Plots from Boosted Regression Tree models using development data set for each of the three invertebrate metrics (EPTR (Richness); Intolerant Taxa (Intol_rich) and NonInsect Richness (NONINSR)) for each region that were not shown in the full paper in PlosOne.**

**Full Region** -- Plots in order from left to right: 1) Percent Watershed Urban, 2) % Riparian Slope, 3) Average December Runoff (mm), 4) % Riparian Canopy Cover, 5) % Riparian Forest, and 6) % Manmade Stream Channels for EPT richness. Boosted Regression model variable importance values in parentheses.

**Full Region** Plots in order from left to right: 1) Percent Riparian Forest, 2) Watershed Population Density (#/km^2^), 3) Percent Watershed Forest, 4) % Riparian Maximum Elevation (m), and 5) Average Maximum Monthly Runoff (mm) for Intolerant taxa richness. Boosted Regression model variable importance values in parentheses.

**Full Region** Plots in order from left to right: 1) Average March Runoff (mm), 2) % Manmade Stream Channels, 3) Mean Elevation in Watershed, and 4) Mean Slope in Riparian for NonInsect richness. Boosted Regression model variable importance values in parentheses.

**North Central Appalachians** Plots in order from left to right: 1) Minimum April Runoff (mm), 2) Riparian Population Density (#/km^2^), 3) Mean Riparian Canopy, and 4) Watershed Road Density (km/km^2^) for EPT taxa richness. Boosted Regression model variable importance values in parentheses.

**North Central Appalachians** Plots in order from left to right: 1) % Riparian Forest, 2) Mean Slope in Riparian, 3) Soil Infiltration D (very slow rate), 4) Minimum April runoff (mm), 5) % Riparian Agriculture , 6) Riparian Population Density (#/km^2^), and 7) Watershed Road Density (km/km^2^) for Intolerant taxa richness. Boosted Regression model variable importance values in parentheses.

**North Central Appalachians** Plots in order from left to right: 1) Percent Riparian Forest, 2) Watershed Population Density (#/km^2^), 3) Percent Manmade Channels, 4) Watershed Dam Density (#/km^2^), and 5) Riparian Population Density (#/km^2^) for NonInsect taxa richness. Boosted Regression model variable importance values in parentheses.

**Ridge and Valley** Plots in order from left to right: 1) Percent Riparian Forest, 2) Percent Watershed Agriculture + Urban, 3) Watershed Population Density (#/km^2^), 4) Maximum Monthly Runoff CV, and 5) Percent Watershed Urban for EPT taxa richness. Boosted Regression model variable importance values in parentheses.

**Ridge and Valley** Plots in order from left to right: 1) Percent Riparian Forest, 2) Watershed Population Density (#/km^2^), 3) Percent Watershed Urban, 4) Mean Riparian Canopy, and 5) Mean Watershed Slope for Intolerant taxa richness. Boosted Regression model variable importance values in parentheses.

**Ridge and Valley** Plots in order from left to right: 1) Maximum March Runoff (mm), 2) Maximum May Runoff (mm), 3) Soil Infiltration C (slow rate), and 4) Percent Manmade Channels for NonInsect richness. Boosted Regression model variable importance values in parentheses.

**Northeastern Highlands** Plots in order from left to right: 1) Percent Watershed Urban, 2) Percent Watershed Wetlands, 3) Percent Manmade Channels, and 4) Percent Riparian Forest for EPT taxa richness. Boosted Regression model variable importance values in parentheses.

**Northeastern Highlands** Plots in order from left to right: 1) Percent Watershed Urban, 2) Average July Runoff (mm), 3) Percent Riparian Forest, and 4) Percent Manmade Channels for Intolerant taxa richness. Boosted Regression model variable importance values in parentheses.

**Northeastern Highlands** Plots in order from left to right: 1) Maximum January Runoff (mm), 2) Average March Runoff (mm), 3) Percent Riparian Wetlands, 4) Mean Slope in Riparian, and 5) Watershed Population Density (#/km^2^) for NonInsect taxa richness. Boosted Regression model variable importance values in parentheses.

**Northern Piedmont** Plots in order from left to right: 1) Percent Watershed Urban, 2) Watershed Mean Elevation (m), 3) Percent Riparian Forest, and 4) Percent Manmade Channels for EPT taxa richness. Boosted Regression model variable importance values in parentheses.

**Northern Piedmont** Plots in order from left to right: 1) Percent Watershed Urban, 2) Riparian Maximum Elevation (m), 3) Mean Watershed Slope, 4) Soil Infiltration B (moderate rate), 5) % Riparian Wetland, and 6) Watershed Population Density (#/km^2^), for Intolerant taxa richness. Boosted Regression model variable importance values in parentheses.

**Northern Piedmont** Plots in order from left to right: 1) Maximum April Runoff (mm), 2) Maximum Riparian Elevation (m), 3) Soil Infiltration C (slow rate), and 4) Percent Manmade Channels for NonInsect richness. Boosted Regression model variable importance values in parentheses.
