# Supplementary material for: Stream Macroinvertebrate Response Models for Bioassessment Metrics: Addressing the Issue of Spatial Scale
Source: PLoS One. 2014 Mar 27;9(3):e90944. doi: 10.1371/journal.pone.0090944 (PMC3968005; doi:10.1371/journal.pone.0090944)
Supplement: Appendix S1 — List of GIS data sets and their sources. (DOCX) [file pone.0090944.s003.docx]

**Appendix 1.** GIS datasets and their sources.*

| \| Spatial Dataset \| Data Source \| Source Data format \| Processing format \| Resolution/Scale \| Reference \| \| --- \| --- \| --- \| --- \| --- \| --- \| \| Ecoregions \| EPA Omernik's Level  III Ecoregions \| Vector \| Vector \| 1:7,500,000 \| U.S Environmental Protection Agency Dataset, Digital data  Available at URL: http://www.epa.gov/wed/pages/ecoregions/level_iii_iv.htm \| \| Elevation \| National Elevation  Dataset (NED) \| Raster \| Raster \| 10 meter \| U.S. Geological Survey, National Elevation Dataset, Digital data, Available at URL: http://nationalmap.gov/ \| \| Slope \| National Elevation  Dataset (NED) \| Raster \| Raster \| 10 meter \| U.S. Geological Survey, National Elevation Dataset, Digital data, Available at URL: http://nationalmap.gov/ \| \| Land Cover 2001 \| National Land Cover Dataset 2001(NLCD) \| Raster \| Raster \| 30 meter \| U.S. Geological Survey, National Land Cover Dataset 2001, Digital  data, Available at URL: http://www.mrlc.gov/ \| \| Road Networks \| U.S. Census Bureau  Tiger \| Vector \| Vector \| 1:100,000 \| U.S. Census Bureau, TIGER line data, Digital data, Available at  URL: http://www.census.gov/geo/www/tiger/ \| \| Soil Infiltration  Capacity \| USDA NRCS  STATSGO \| Vector \| Vector \| 1:250,000 \| Natural Resource Conservation Service, STATSGO soils data,  Digital data, Available at URL: http://datagateway.nrcs.usda.gov/ \| \| Hydrography \| National Hydrography Dataset (NHD) \| Vector \| Vector \| 1:24,000 \| U.S. Geological Survey, National Hydrography Dataset, Digital  data, Available at URL: http://nhd.usgs.gov/data.html \| \| Dams \| National Inventory of Dams \| Vector \| Vector \| Various \| U.S. Army Corps of Engineers, National Inventory of Dams, Digital  data, Not publicly available \| \| Pollution Point  Sources \| EPA Toxic Release Inventory \| Vector \| Vector \| Various \| U.S. Environmental Protection Agency, Toxic Release Inventory  data, Digital data, Available at URL: http://www.epa.gov/tri/tridata/tri05/data \| \| Precipitation \| Oregon State University PRISM \| Raster \| Raster \| 30 arc-second \| PRISM Group, Oregon State University, Precipitation data for the  U.S., Digital data, Available at URL: http://www.prismclimate.org \| \| Population Density \| U.S. Census Bureau Census 2000 \| Vector \| Vector \| 30 meter \| U.S. Census Bureau, Census 2000, Digital data, Available at URL: http://www.census.gov/main/www/cen2000.html \| \| Canopy Cover \| National Land Cover Dataset 2001 Percent  Tree Canopy \| Raster \| Raster \| 30 meter \| U.S. Geological Survey, National Land Cover Dataset 2001,  Percent Tree Canopy (Version 1.0) Dataset. Digital Data. Available  at URL: http://www.mrlc.gov \| |
| --- | --- | --- | --- | --- | --- | --- | --- | --- | --- | --- | --- | --- | --- | --- | --- | --- | --- | --- | --- | --- | --- | --- | --- | --- | --- | --- | --- | --- | --- | --- | --- | --- | --- | --- | --- | --- | --- | --- | --- | --- | --- | --- | --- | --- | --- | --- | --- | --- | --- | --- | --- | --- | --- | --- | --- | --- | --- | --- | --- | --- | --- | --- | --- | --- | --- | --- | --- | --- | --- | --- | --- | --- | --- | --- | --- | --- | --- | --- |

* TIGER–Topologically Integrated Geographic Encoding and Referencing, PRISM– Parameter-elevation Regressions on Independent Slopes Model, STATSGO–State Soil Geographic data base.
