# Supplementary material for: Stream Macroinvertebrate Response Models for Bioassessment Metrics: Addressing the Issue of Spatial Scale
Source: PLoS One. 2014 Mar 27;9(3):e90944. doi: 10.1371/journal.pone.0090944 (PMC3968005; doi:10.1371/journal.pone.0090944)
Supplement: Supplementary file 3 [file pone.0090944.s002.docx]

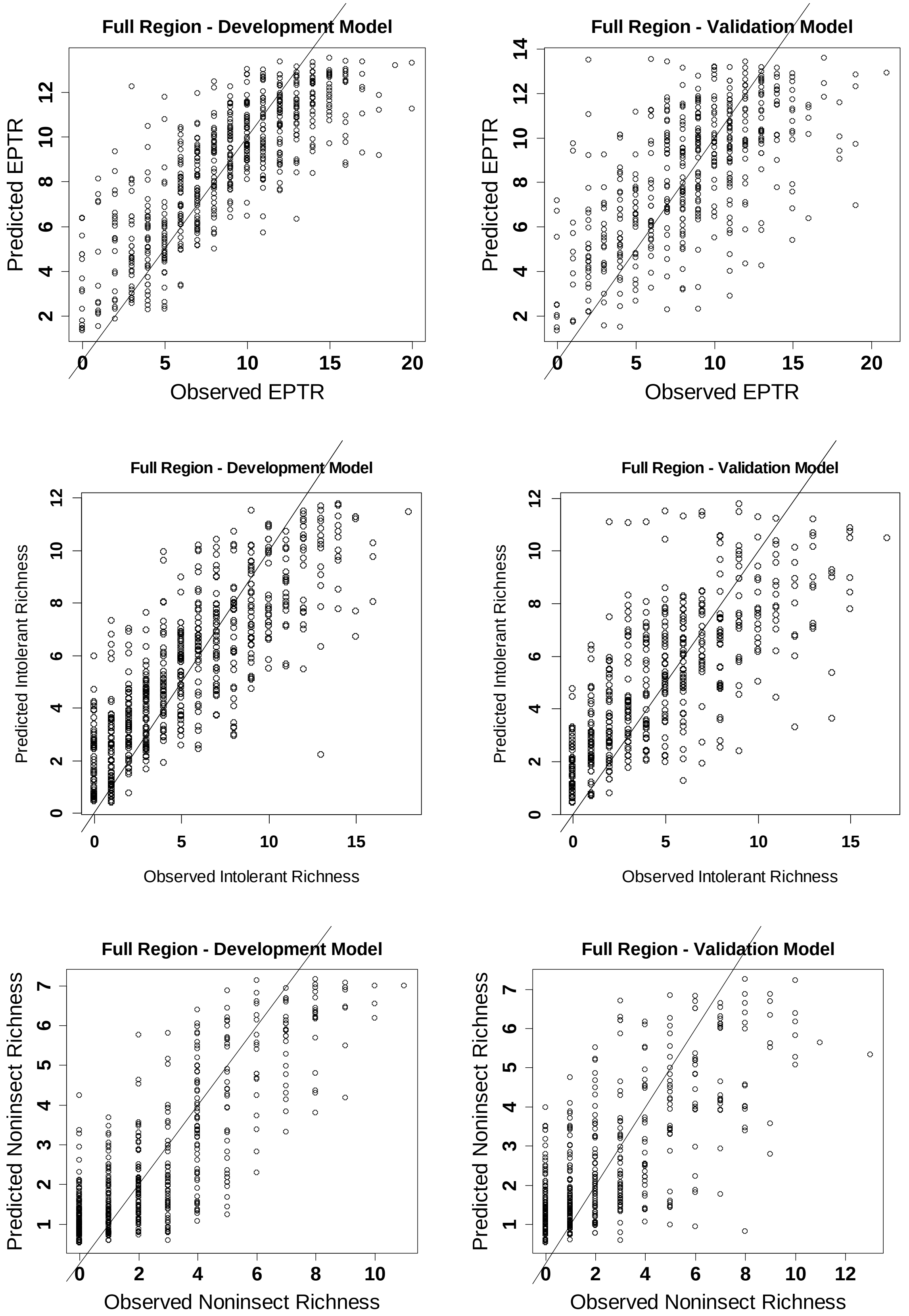
Full Region - Development Model
Predicted EPTR
Observed EPTR
Full Region - Validation Model
Predicted EPTR
Observed EPTR
Full Region - Development Model
Predicted Intolerant Richness
Observed Intolerant Richness
Full Region - Validation Model
Predicted Intolerant Richness
Observed Intolerant Richness
Full Region - Development Model
Predicted Noninsect Richness
Observed Noninsect Richness
Full Region - Validation Model
Predicted Noninsect Richness
Observed Noninsect Richness

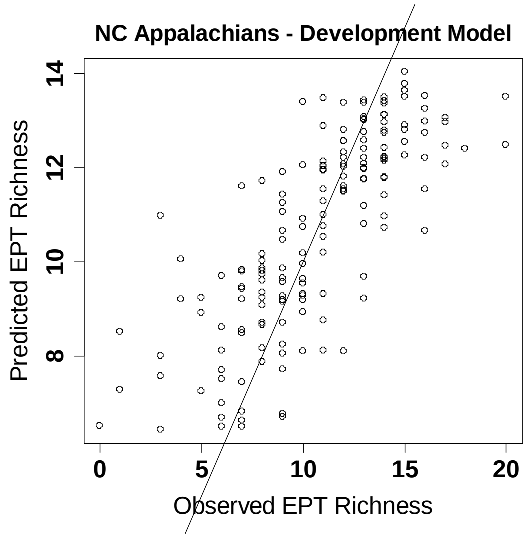
NC Appalachians - Development Model
Predicted EPT Richness
8
10
12
14
0
5
10
15
20
Observed EPT Richness

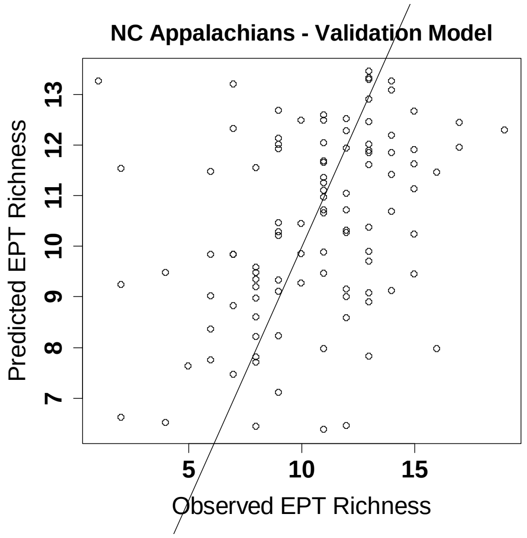
NC Appalachians - Validation Model
Predicted EPT Richness
7
8
9
10
11
12
13
5
10
15
Observed EPT Richness

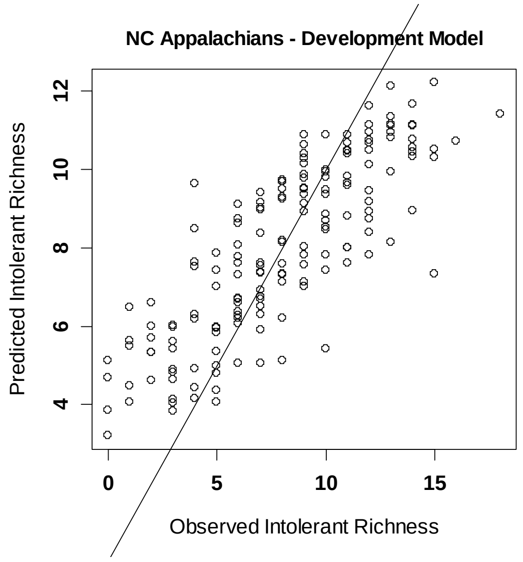
NC Appalachians - Development Model
Predicted Intolerant Richness
4
6
8
10
12
0
5
10
15
Observed Intolerant Richness

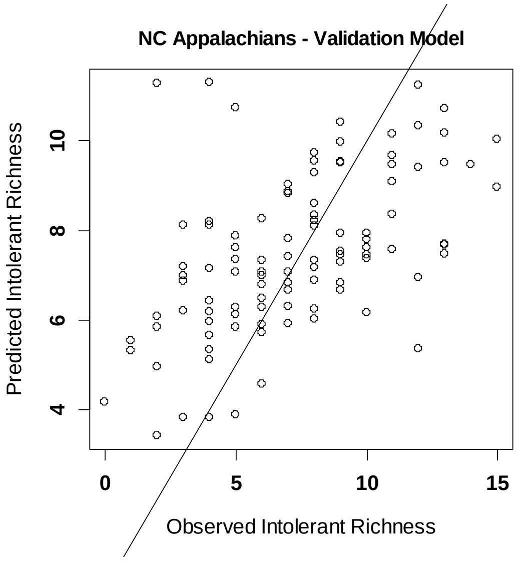
NC Appalachians - Validation Model
Predicted Intolerant Richness
4
6
8
10
0
5
10
15
Observed Intolerant Richness

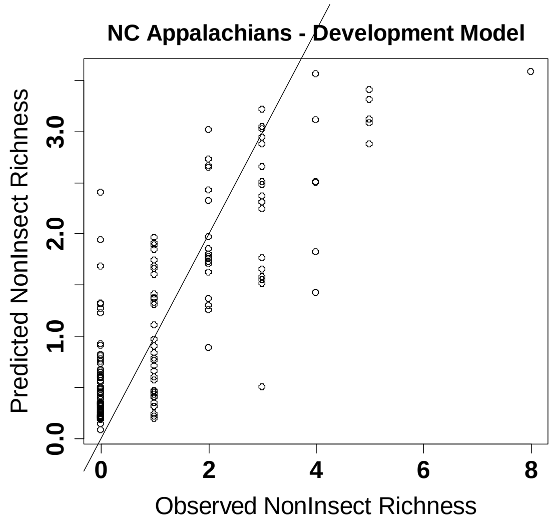
NC Appalachians - Development Model
Predicted NonInsect Richness
0.0
1.0
2.0
3.0
0
2
4
6
8
Observed NonInsect Richness

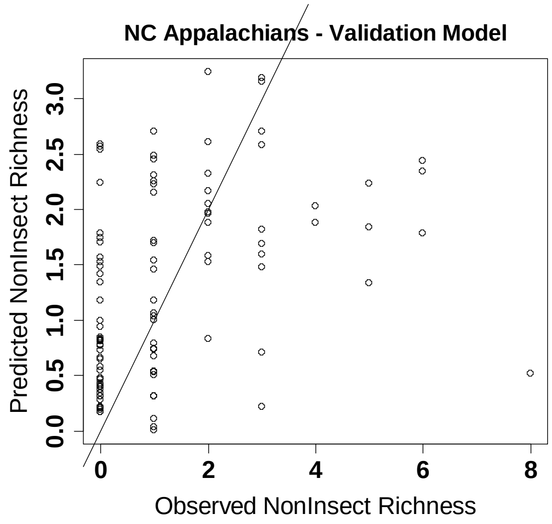
NC Appalachians - Validation Model
Predicted NonInsect Richness
0.0
0.5
1.0
1.5
2.0
2.5
3.0
0
2
4
6
8
Observed NonInsect Richness

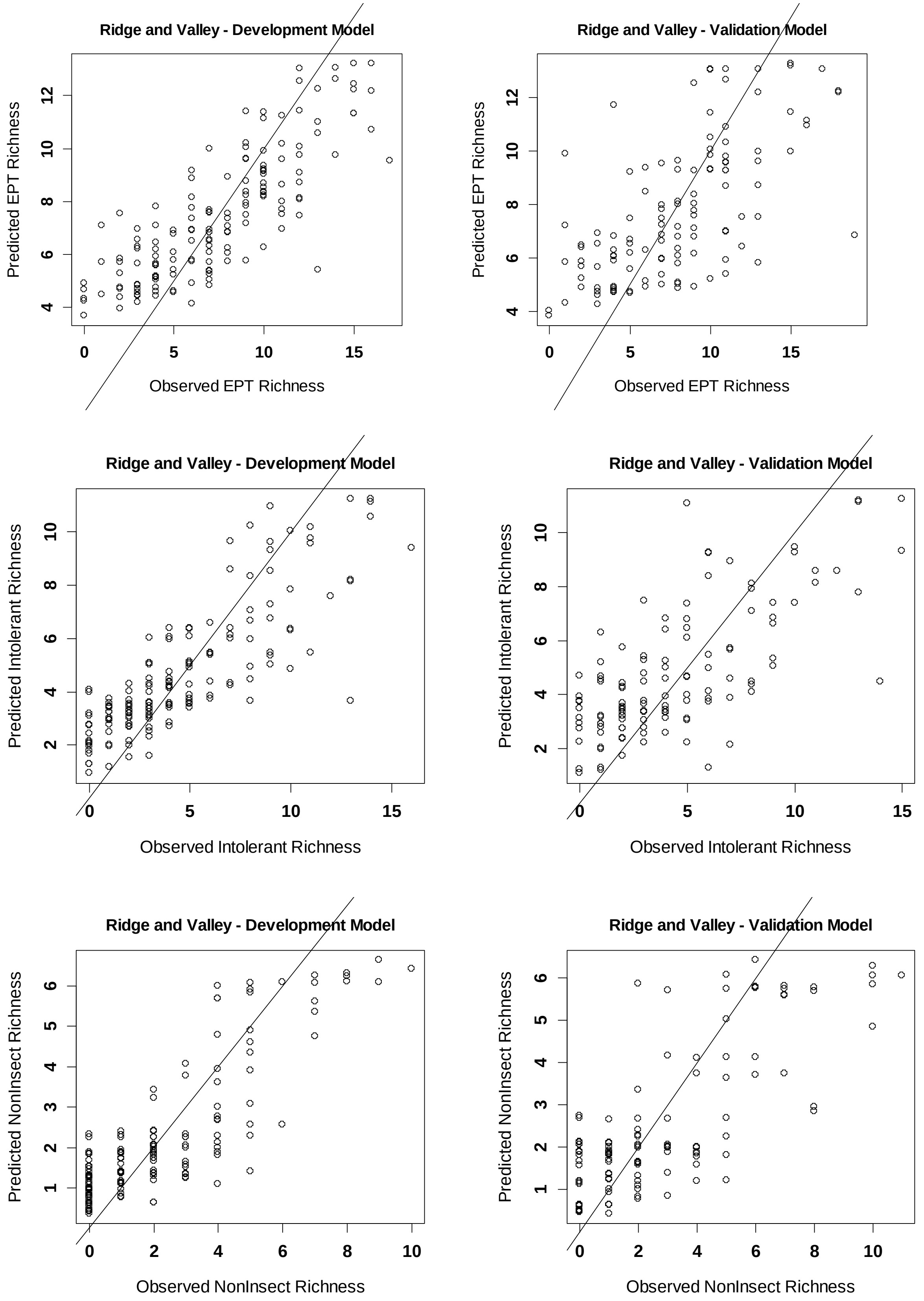
Ridge and Valley - Development Model
Predicted EPT Richness
4 6 8 10 12
0 5 10 15
Observed EPT Richness
Ridge and Valley - Validation Model
Predicted EPT Richness
4 6 8 10 12
0 5 10 15
Observed EPT Richness
Ridge and Valley - Development Model
Predicted Intolerant Richness
2 4 6 8 10
0 5 10 15
Observed Intolerant Richness
Ridge and Valley - Validation Model
Predicted Intolerant Richness
2 4 6 8 10
0 5 10 15
Observed Intolerant Richness
Ridge and Valley - Development Model
Predicted NonInsect Richness
1 2 3 4 5 6
0 2 4 6 8 10
Observed NonInsect Richness
Ridge and Valley - Validation Model
Predicted NonInsect Richness
1 2 3 4 5 6
0 2 4 6 8 10
Observed NonInsect Richness

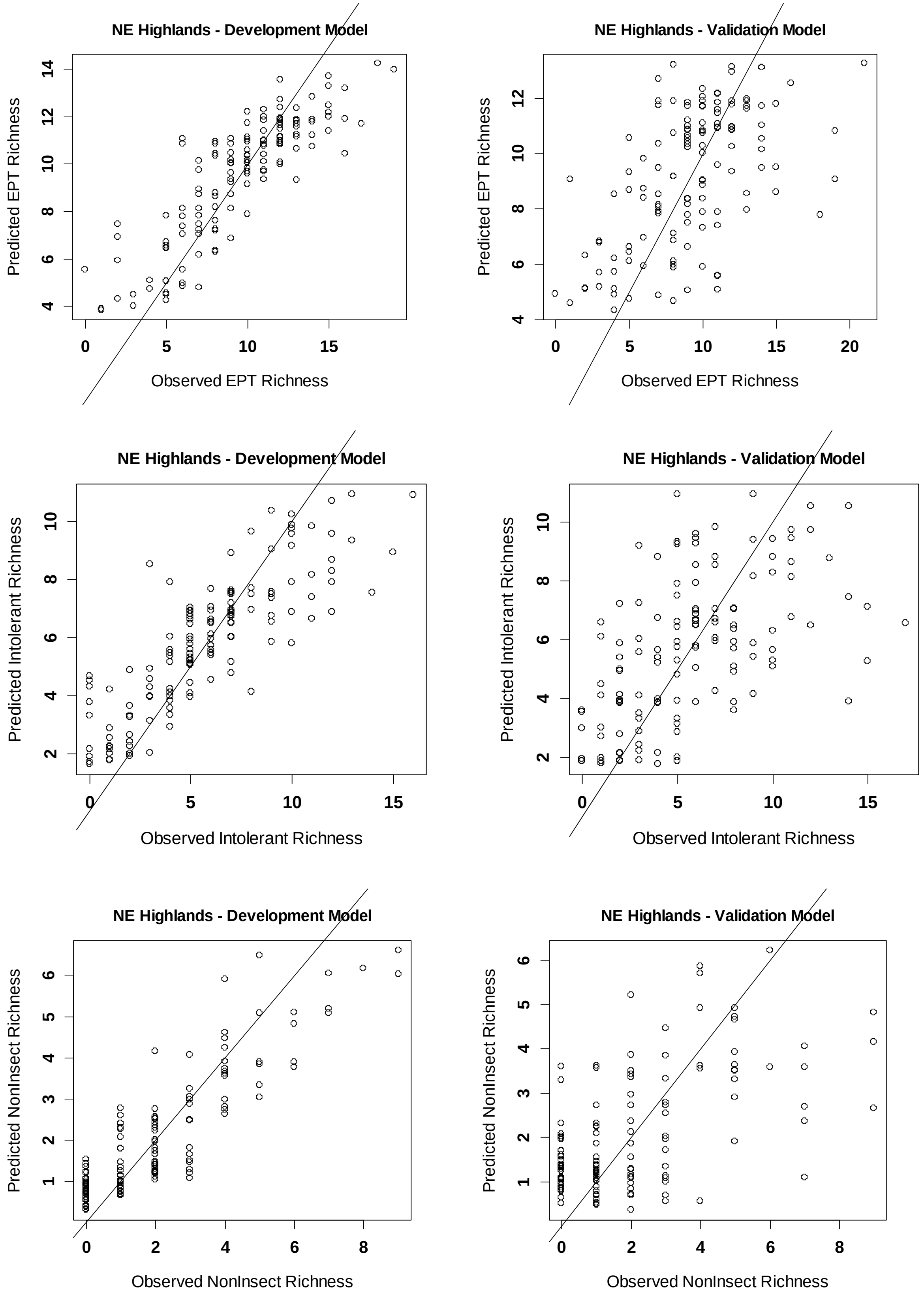
NE Highlands - Development Model
Predicted EPT Richness
Observed EPT Richness
NE Highlands - Validation Model
Predicted EPT Richness
Observed EPT Richness
NE Highlands - Development Model
Predicted Intolerant Richness
Observed Intolerant Richness
NE Highlands - Validation Model
Predicted Intolerant Richness
Observed Intolerant Richness
NE Highlands - Development Model
Predicted NonInsect Richness
Observed NonInsect Richness
NE Highlands - Validation Model
Predicted NonInsect Richness
Observed NonInsect Richness

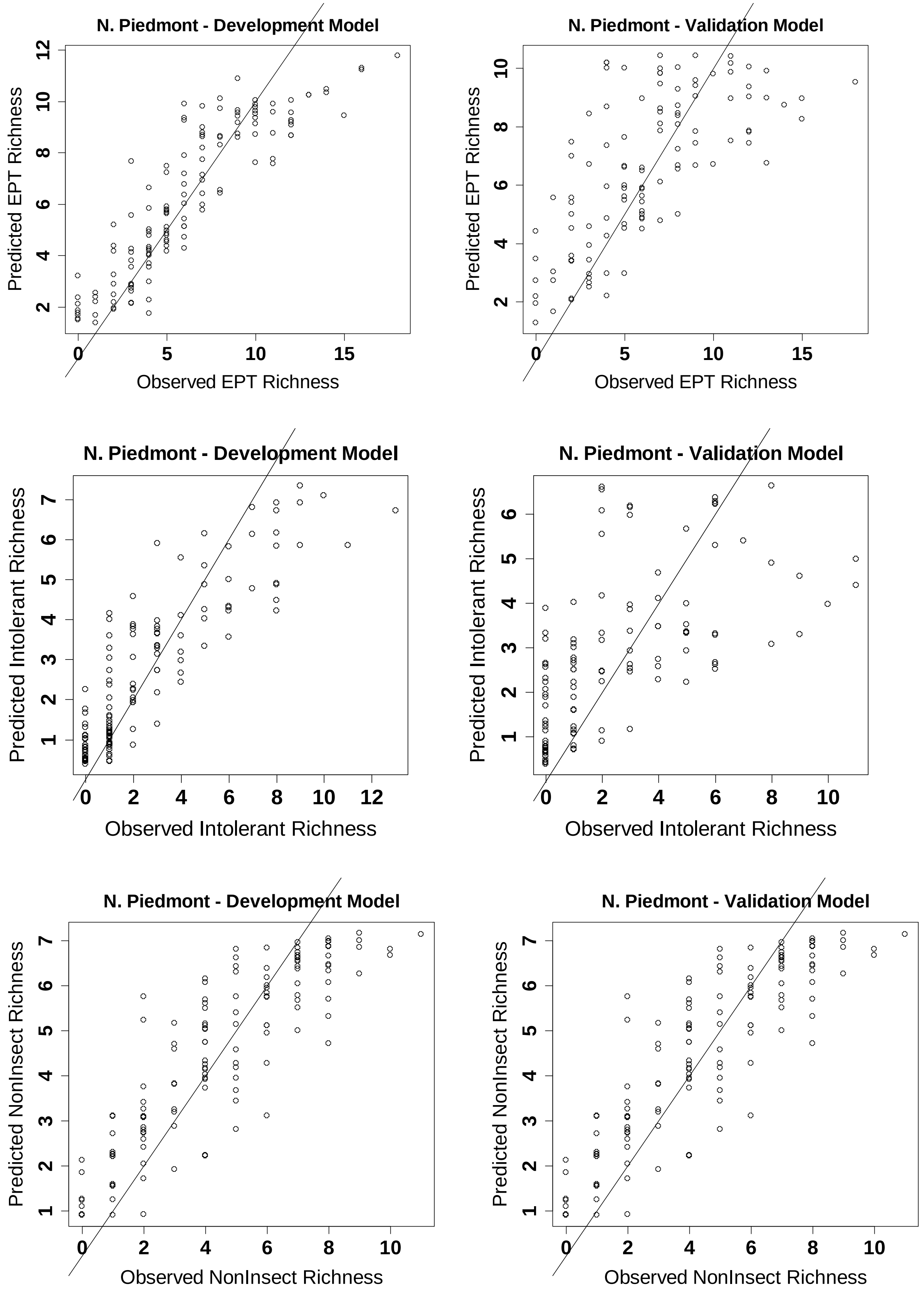
N. Piedmont - Development Model
Predicted EPT Richness
Observed EPT Richness
N. Piedmont - Validation Model
Predicted EPT Richness
Observed EPT Richness
N. Piedmont - Development Model
Predicted Intolerant Richness
Observed Intolerant Richness
N. Piedmont - Validation Model
Predicted Intolerant Richness
Observed Intolerant Richness
N. Piedmont - Development Model
Predicted NonInsect Richness
Observed NonInsect Richness
N. Piedmont - Validation Model
Predicted NonInsect Richness
Observed NonInsect Richness
